# DNA binding specificities of the long zinc-finger recombination protein PRDM9

**DOI:** 10.1186/gb-2013-14-4-r35

**Published:** 2013-04-24

**Authors:** Timothy Billings, Emil D Parvanov, Christopher L Baker, Michael Walker, Kenneth Paigen, Petko M Petkov

**Affiliations:** 1 Center for Genome Dynamics, The Jackson Laboratory, 600 Main Street, Bar Harbor, ME 04609, USA; 2 National Centre for Biomolecular Research and Department of Biology, Masaryk University, Brno 625 00, Czech Republic

**Keywords:** recombination hotspots, PRDM9, DNA binding, EMSA, zinc-finger proteins

## Abstract

**Background:**

Meiotic recombination ensures proper segregation of homologous chromosomes and creates genetic variation. In many organisms, recombination occurs at limited sites, termed 'hotspots', whose positions in mammals are determined by PR domain member 9 (PRDM9), a long-array zinc-finger and chromatin-modifier protein. Determining the rules governing the DNA binding of PRDM9 is a major issue in understanding how it functions.

**Results:**

Mouse PRDM9 protein variants bind to hotspot DNA sequences in a manner that is specific for both PRDM9 and DNA haplotypes, and that *in vitro *binding parallels its *in vivo *biological activity. Examining four hotspots, three activated by *Prdm9^Cst ^*and one activated by *Prdm9^Dom2^*, we found that all binding sites required the full array of 11 or 12 contiguous fingers, depending on the allele, and that there was little sequence similarity between the binding sites of the three *Prdm9^Cst ^*activated hotspots. The binding specificity of each position in the Hlx1 binding site, activated by *Prdm9^Cst^*, was tested by mutating each nucleotide to its three alternatives. The 31 positions along the binding site varied considerably in the ability of alternative bases to support binding, which also implicates a role for additional binding to the DNA phosphate backbone.

**Conclusions:**

These results, which provide the first detailed mapping of PRDM9 binding to DNA and, to our knowledge, the most detailed analysis yet of DNA binding by a long zinc-finger array, make clear that the binding specificities of PRDM9, and possibly other long-array zinc-finger proteins, are unusually complex.

## Background

Genetic recombination is an essential feature of meiosis, assuring an appropriate segregation of chromatids at the first meiotic division, and generating an evolutionarily important source of genetic variation by providing new arrangements of alleles between genes linked on the same chromosome. In many organisms, notably yeast [1], higher plants [2], and mammals including humans and mice [3-5], recombination is concentrated along chromosomes at limited sites known as 'hotspots'. Typically a kilobase in extent, hotspots are surrounded by long stretches of DNA, tens to hundreds of kilobases in extent, that are essentially devoid of recombination in humans and mice.

Recently, several groups have shown that PR domain member 9 (PRDM9), a zinc-finger (ZF) protein with histone 3 lysine 4 (H3K4) methyltransferase activity, plays a key role in determining the locations of hotspots in both mice and humans [6-8]. It is presently proposed that PRDM9 binds to appropriate DNA sequences in meiotic chromatids, generates activated chromatin by virtue of its H3K4 methyltransferase activity, and somehow guides the generation of double-strand breaks (DSBs) at those sites by the topoisomerase-like protein SPO11 [9]. Analyses of individual human hotspots and a number of genome-wide studies have implicated PRDM9 as the predominant regulator of hotspot placement [6,7,10,11]. In mice, analyses of genome-wide hotspots of DSB formation [12] make it clear that PRDM9 determines the location of virtually all hotspots, with the clear exception of the obligate crossover at the pseudoautosomal region, at which recombination occurs equally well with different variants of PRDM9 present, or indeed no variant at all. There is also evidence that *Prdm9 *participates in transcriptional regulation [13,14], which may be related to its involvement in hybrid sterility [14].

Although PRDM9 plays a very important role in mammalian recombination, there is considerable uncertainty as to how it physically determines hotspot locations and then directs DSB formation there rather than at other trimethylated H3K4 (H3K4-me3) sites, of which there are many. Meeting these challenges has repercussions both for expanding the current understanding of recombination biology and for the insights this could provide in understanding the functions of other regulatory proteins with ZF arrays. More than 4% of human protein-coding genes contain ZF arrays, and half of those arrays are comparable in size with those present in PRDM9 [15-17].

The identification of PRDM9 as a regulator of human recombination [7] relied on the finding that the DNA sequence predicted to bind to the ZF array of the most common human variant (variant A) matched a 13 bp consensus sequence that characterizes 41% of human hotspots [18], and the fact that although this sequence is present in chimpanzee DNA, it does not characterize chimpanzee hotspots. Baudat *et al. *[6] correlated human hotspot activity with human allelic variation at PRDM9, and predicted a mouse PRDM9 DNA binding sequence found in a mouse hotspot. Parvanov *et al. *[8] identified *Prdm9 *in mice by genetically mapping the gene controlling hotspot activity to a 181 Kb interval containing four genes, three of which could be excluded as candidates.

Although PRDM9 reportedly binds to hotspot DNA [6,19], there is still considerable confusion about the nature of the DNA sequences recognized by the ZF array of PRDM9 and how this protein achieves its locational specificity. Many more copies of the human consensus sequence are found in the genome at non-hotspot sites than at the hotspots themselves [18], and Berg *et al. *[10] showed that human hotspots possessing or not possessing this consensus sequence are equally dependent on PRDM9. In mice, the consensus sequence originally predicted for the mouse PRDM9^Cst ^variant [6] is more commonly present in non-hotspot than in hotspot regions, and the ZF prediction programs used in various studies [20-22] also predict that the mouse Dom2 variant of PRDM9 (PRDM9^Dom2^) should bind to hotspots that genetic studies have shown are not activated by this allele. Nevertheless, when tested experimentally, longer oligonucleotides containing buried sequences matching the predicted recognition motifs of two human *PRDM9 *alleles have been shown to bind PRDM9 protein expressed in cell cultures [6,19].

To address the DNA binding problem experimentally, we expressed the mouse *Prdm9^Cst ^*allele (from the CAST/EiJ strain; referred to below as CAST) and the *Prdm9^Dom2 ^*allele (from the C57BL/6J strain; referred to below as B6) in *Escherichia coli*. and determined the abilities of their respective protein products to bind DNA sequences from three mouse hotspots (Hlx1, Esrrg1, and Psmb9) known from genetic evidence to be activated by *Pdrm9^Cst ^*and one (Pbx1) known to be activated by *Prdm9^Dom2^*. Binding between expressed PRDM9 and DNA was tested both by the variant-specific ability of PRDM9 to modify the electrophoretic mobility of target DNA sequences and conversely, by the ability of DNA binding-site sequences to physically sequester PRDM9.

## Results

### Histone H3K4 trimethylating activity

As an indication that the PRDM9 expressed in *E. coli *is native protein, we first confirmed that it retains its histone trimethylating activity. Both the induced PRDM9^Dom2 ^and the PRDM9^Cst ^*E. coli *extracts showed enhanced H3K4 trimethylating activity compared with uninduced extracts, and induced empty vector (see Additional file 1, Figure S1).

### Fine mapping of Prdm9^Dom2 ^and PRDM9^Cst ^binding sites

The Hlx1 and Esrrg-1 hotspots, described in our previous work [8,23], require activation by the *Prdm9^Cst ^*allele, as does Psmb9 [6]. We concluded that the Pbx1 hotspot is dependent on the *Prdm9^Dom2 ^*allele because it was only active in crosses involving B6 and not active in crosses lacking this genetic background (see Additional file 2, Figure S2A).

**Figure 1:**
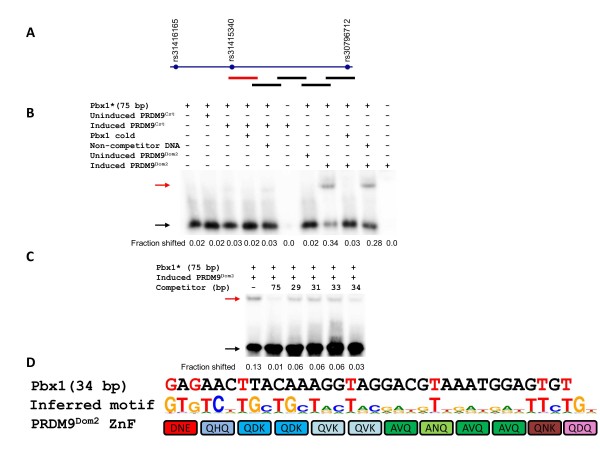
**Mapping the PRDM9^Dom2 ^binding site of Pbx1**. **(A) **Scheme of Pbx1 hotspot with polymorphisms and amplicons for initial testing of PRDM9^Dom2 ^binding. The red line shows the position of the single binding site detected at this hotspot. **(B) **Pbx1 specifically binds to PRDM9^Dom2 ^but not PRDM9^Cst^. The compositions of the binding reactions are shown above each lane. Red arrow, shifted band; black arrow, unbound fragment. **(C) **Detailed mapping of the Pbx1 binding site. The compositions of the binding reactions are shown above each lane. For the sequences of the competitor oligos used for final mapping, see Additional file 11 (Additional experimental procedures). Red arrow, shifted band; black arrow, unbound fragment. **(D) **The sequence of the 34 bp oligo representing the minimal binding site is aligned to the inferred PRDM9^Dom2 ^binding motif [6,21] and the zinc-finger array of PRDM9^Dom2 ^(amino acids at positions -1, 3, and 6 relative to the α-helix of each finger are shown). The strong matches of the binding site to the motif are in red.

Pbx1 had a single, PRDM9^Dom2 ^allele-specific binding site located at the left end of the interval tested (Figures 1A, B; see Additional file 2, Figure S2B). The shortest Pbx1 oligo that showed binding to this allele, which has 12 contiguous ZFs, was 34 bp long (Figure 1C; see Additional file 2, Figure S2C). This is close to the 36 bp that is predicted if all of the ZFs bind their expected 3 bp sections. The binding site contained seven strong matches to the binding site predicted for PRDM9^Dom2 ^by the linear Persikov-Singh algorithm [22] when aligned to fingers 1 to 11 (Figure 1D), counting the 15 nucleotides that are found at > 60% frequency at each position. However, this was not statistically significant compared with the 3.75 matches expected by chance (*P *= 0.057 by a binomial distribution). The Pbx1 binding site is positioned 6 bp distal to the single-nucleotide polymorphism (SNP) between the B6 and CAST strains within the hotspot (see Additional file 2, Figure S2D). Surprisingly, although this hotspot has a single binding site, the locations of the genetic crossovers within this hotspot depend on the nature of the genetic cross used in its detection. In the case of the B6xCAST and B6xPWD/PhJ interstrain crosses, where the recombining mice are heterozygous across the entire genome, genetic crossovers were distributed on both sides of the binding site. However, in the case of the B6xB6. CAST-1T congenic cross, where the F1 mice are heterozygous only for the distal 100 Mb of chromosome 1 and are homozygous across the rest of the genome, genetic crossovers were located to one side of the binding site (see Additional file 2, Figure S2A).

**Figure 2:**
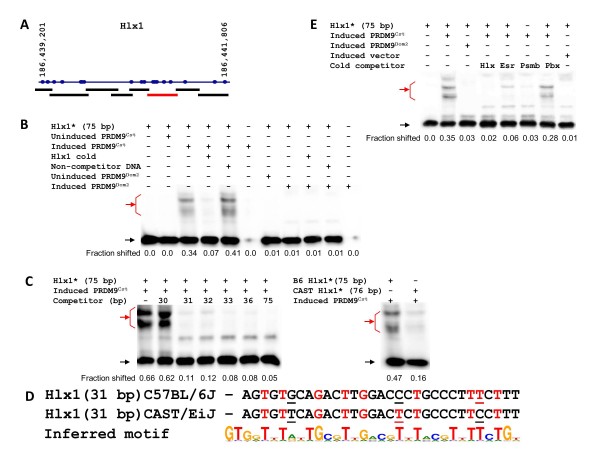
**Defining the PRDM9^Cst ^binding site of Hlx1**. **(A) **Scheme of Hlx1 hotspot with polymorphisms between the C57BL/6J (B6) and the CAST/EiJ (CAST) mouse strains (from Paigen *et al. *[5]) and amplicons for initial testing of PRDM9^Cst ^binding. The positions of single-nucleotide polymorphisms (SNPs) were taken from the National Center for Biotechnology Information (NCBI) build 37. The red line represents the only fragment showing binding at Hlx1. **(B) **Hlx1 specifically binds to PRDM9^Cst ^but not PRDM9^Dom2^. The compositions of the binding reactions are shown above each lane. Red arrow, shifted band; black arrow, unbound fragment. **(C) **Detailed mapping of the Hlx1 binding site. The compositions of the binding reactions are shown above each lane. For the sequences of the competitor oligos used for final mapping, see Additional file 11 (Additional experimental procedures). (Left panel) Definition of the minimal binding site; (right panel) Strength of binding of PRDM9^Cst ^to B6 and CAST sequences of the Hlx1 binding site. Red arrow, shifted band; black arrow, unbound fragment. **(D) **Sequences of the Hlx1 minimal binding sites in B6 and CAST strains aligned to the inferred PRDM9^Cst ^binding motif. Sequence differences are underlined. The strong matches of the binding sites to the motif are in red. **(E) **Hlx1 competes with the other PRDM9^Cst^-dependent binding sites. The compositions of the binding reactions are shown above each lane. Red arrow, shifted band; black arrow, unbound fragment.

Hlx1 also possesses a single, variant-specific binding site, this time located at the middle of the genetic interval defining the hotspot (Figure 2A, B; see Additional file 3, Figure S3A). The shortest Hlx1 oligo showing binding to PRDM9^Cst ^was 31 bp long (Figure 2C, left panel), close to the 33 bp predicted if all 11 contiguous ZFs in this allele bind their expected 3 bp. Both B6 and CAST sequences showed nine matches to the binding site predicted for PRDM9^Cst ^by the linear Persikov-Singh algorithm, but at different positions, and this was significant (*P *= 0.00034) compared with the 3.5 matches expected by chance.

The B6 and CAST sequences at the Hlx1 binding site differ at three positions (Figure 2D; see Additional file 3, Figure S3B), of which two out of three affect binding affinity and crossover rate in parallel at this hotspot. Although both the B6 and CAST sequences bound PRDM9^Cst^, binding by the B6 sequence was about 2.9 times stronger than that by the CAST sequence (Figure 2C, right panel). This corresponds with our previous demonstration that initiation of meiotic recombination at Hlx1 is about 2.5 times more frequent on the B6 chromosome than on the CAST chromosome [5]. A recent estimate of Hlx1 by another method found about a four-fold difference between the B6 and CAST sequences using 41 bp oligonucleotides [19].

**Figure 3:**
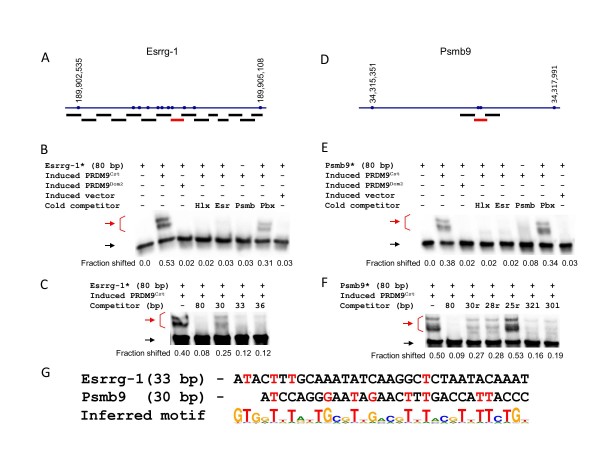
**Defining the PRDM9^Cst ^binding sites of Esrrg-1 and Psmb9**. **(A) **Scheme of Esrrg-1 hotspot with polymorphisms between C57BL/6J (B6) and CAST/EiJ (CAST) and amplicons for initial testing of PRDM9^Cst ^binding. The red line represents the only fragment showing binding at this hotspot. **(B) **Esrrg-1 specifically binds to PRDM9^Cst ^but not PRDM9^Dom2 ^and competes with the other PRDM9^Cst^-dependent binding sites. The compositions of the binding reactions are shown above each lane. Red arrow, shifted band; black arrow, unbound fragment. **(C) **Detailed mapping of the Esrrg-1 binding site. The compositions of the binding reactions are shown above each lane. For the sequences of the competitor oligos used for final mapping, see Additional file 11 (Additional experimental procedures). Red arrow, shifted band; black arrow, unbound fragment. **(D) **Scheme of Psmb9 hotspot with polymorphisms between B6 and CAST, and amplicons for initial testing of PRDM9^Cst ^binding. The approximate position of the Psmb9 binding site was reported previously [19], and therefore only three amplicons surrounding it were tested. Only the middle fragment showed binding to PRDM9^Cst^. **(E) **Psmb9 specifically binds to PRDM9^Cst ^but not PRDM9^Dom2 ^and competes with the other PRDM9^Cst^-dependent binding sites. The compositions of the binding reactions are shown above each lane. Red arrow, shifted band; black arrow, unbound fragment. **(F) **Detailed mapping of the Psmb9 binding site. The compositions of the binding reactions are shown above each lane. For the sequences of the competitor oligos used for final mapping, see Additional file 11 (Additional experimental procedures). Red arrow, shifted band; black arrow, unbound fragment. **(G) **Sequences of the Esrrg-1 and Psmb9 minimal binding sites aligned with the inferred PRDM9^Cst ^binding motif. The strong matches of the binding sites to the motif are in red.

Esrrg-1 had a single, variant-specific binding site, also located near the middle of the genetic interval identifying this hotspot (Figure 3A; see Additional file 4, Figure S4A). This binding site lacked any sequence differences between B6 and CAST (see Additional file 4, Figure S4B). The minimum length of the binding site for the Esrrg-1 hotspot was 33 bp (Figure 3C), and there were only four strong matches to the binding site predicted for PRDM9^Cst ^(Figure 3G), which was not different from the chance expectation (*P *= 0.31).

The Psmb9 hotspot has been identified and genetically characterized previously [24]. We tested only its central region, where the PRDM9^Cst ^binding reportedly occurs [19] (Figure 3D), confirming this prediction. Testing the reduced oligos, we found a 30 bp minimal binding site showing allele specificity (Figure 3F). Its best alignment to the predicted site had eight strong matches (Figure 3G), which is likely to be significant (*P *= 0.00098).

### Comparison of PRDM9^Cst ^binding sites

The lengths of the three binding sites (30 to 33 bp) suggests that binding involves all of the Zn fingers in the array, with the possible exception of either the first or the last finger, noting that the first finger has an ECH2 configuration rather than C2H2.

There is very little similarity between the sequences of the three PRDM9^Cst ^binding sites. Any pairwise matches in sequence for the three sites were distributed over the entire length of the binding sites. The best alignment of the three minimal binding sites for the PRDM9^Cst ^ZF array identified only four conserved positions (*P *= 0.052) (see Additional file 5, Figure S5, red); two would have been expected by chance. These triple matches are located at positions predicted to bind the second, fourth, sixth, and eighth fingers of PRDM9^Cst^. Pairwise comparisons identified 15 matches between Hlx1 and Esrrg-1 (see Additional file 5, Figure S5, red and green), 12 matches between Hlx1 and Psmb9 (see Additional file 5, Figure S5, red and yellow), and only 6 matches between Psmb9 and Esrrg-1 (see Additional file 5, Figure S5, red and cyan). We examined this level of similarity using two statistical methods. Using the χ^2 ^test, the probability that the number of nucleotide matches between the three sites (scored as 0, 2, or 3 matches at each position) exceeds chance was 0.048 (barely significant), and this significance entirely disappeared when we considered only the 17 positions where the mutation analysis (see below) indicated the strongest evidence for nucleotide specificity. Additionally, using the binomial distribution for pairwise comparisons, we found some support for similarity between Hlx1 and Esrrg-1 (*P *= 0.003) and between Hlx1 and Psmb9 (P = 0.048), but not between Psmb9 and Esrrg-1 (*P *= 0.75). Given this marginal sequence similarity between the three PRDM9^Cst ^binding sites, we confirmed by testing their ability to compete with each other for binding that they do in fact bind to the same molecular entity. The sites do compete with each other, and the data suggest that Hlx1 and Psmb9 both bind more strongly than Esrrg-1 (Figure 2E; Figure 3B, E). There was also no consistency in the binding specificity of the same ZF located at different positions in the array. The amino acids at positions -1, 2, 3, and 6 in each finger are thought to make contact with the DNA helix and determine binding specificity [21,22,25]. These amino acids are identical (ASNQ), as are all the rest of the amino acids in fingers 2, 5, 7, and 9 of the PRDM9^Cst ^Zn finger array, and all 11 fingers in this array contain serine at the -2 position, which is also thought to contribute to specificity. Nevertheless, fpr the three PRDM9^Cst ^binding sites, there is no consistency in the four triplets that these four fingers bind (see Additional file 5, Figure S5). A similar result was seen for the nucleotides binding the pairs of identical fingers in the PRDM9^Dom2 ^variant at the Pbx1 hotspot.

### *In vivo *H3K4 trimethylation sites

**Figure 4:**
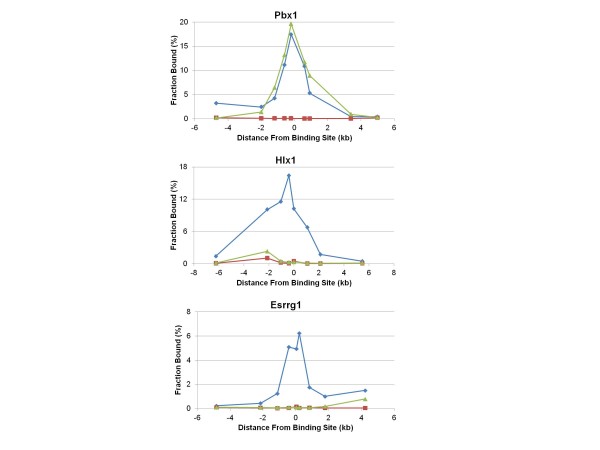
**H3K4-me3 marks are enriched near the binding sites of the hotspot tested**. The peak of H3K4-me3 at hotspots is centered near the PRDM9 binding site. Chromatin was prepared using spermatocytes from mice 12 days post-partum and subjected to chromatin immunoprecipitation (ChIP) with antibody directed to H3K4-me3 or normal rabbit IgG. Quantitative PCR was performed for 8 to 9 amplicons distributed across about 10 kb surrounding the hotspot on immunoprecipitated chromatin and an equal amount of MNase-treated, undiluted input DNA to calculate the fraction of chromatin bound at each amplicon. Blue line, B6 × CAST F1; green line, B6; red line, rabbit IgG (negative control). An identical distribution of H3K4-me3 marks for Psmb9 was shown previously by Grey *et al. *[19].

The locations of the DNA binding sites for all hotspots were within the regions representing the peak locations of meiotic histone H3K4-me3 marks that result from PRDM9 binding; these were measured in mouse testes by chromatin immunoprecipitation (ChIP) using an antibody against H3K4-me3 (Figure 4). The same pattern has been previously reported for Psmb9 [19,26]. It should be noted that the histone modification occurs over appreciable distances, a kilobase or more from the actual binding site itself, indicating that H3K4-trimethylation at hotspots involves nucleosomes beyond those immediately adjacent to the binding site.

### Computer predictions

The algorithms developed by Persikov and Singh [22] are commonly used to predict ZF DNA binding sequences. These include both a linear prediction program based on an elemental identity of trinucleotide binding sequences for each finger, and a polynomial form of the program that takes nucleotide interactions into account. In every case, when both programs were tested against 200 bp sequences surrounding the four identified binding sites, the programs identified multiple binding sites within the 200 bp sequence. In only two of the eight tests was the actual binding site the top scoring site; in two cases, the program failed to identify the binding site at all, and the quality of prediction declined noticeably when tested against longer DNA sequences. The performance of the linear and polynomial prediction programs differed for each hotspot. Only the polynomial program correctly identified the Hlx1 binding site, whereas only the linear program identified Pbx1. Both programs identified Esrrg-1 and Psmb9 (see Additional file 6, Table S1).

As an alternative, we used a position-weighted matrix [27] derived from the detailed binding requirements of the Hlx1 hotspot (see Additional file 7, Table S2) to scan for DNA binding sites that coincide with genetically determined hotspots on mouse chromosomes 1 and 11 [5,28]. Unfortunately, we failed to find a DNA binding motif common to genetically identified hotspots on these chromosomes.

### Mutational characterization of the Hlx1 binding site

**Figure 5:**
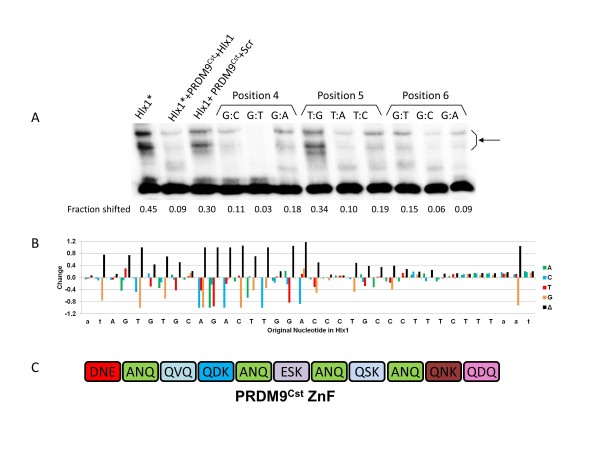
**Nucleotide substitution analysis of Hlx1-B6 binding to PRDM9^Cst^**. **(A) **Competition assay for testing the binding of PRDM9^Cst ^to mutated Hlx1 binding sites. Substitutions of nucleotides 4 to 6; labeled oligos are indicated by asterisk. The compositions of reaction mixtures in the first three lanes are shown above each lane. Lanes 4 to 12 show the competition test with mutated oligos. The position and the nature of each mutation in the oligo are shown. The composition of the reaction mixtures in lanes 4 to 12 is mutated oligo + PRDM9^Cst ^+ oligo* (see Materials and methods). The fraction of the shifted band is indicated below each lane. Red arrow, shifted band; black arrow, unbound fragment. **(B) **Graphic representation of the binding changes at each position produced by the nucleotide substitution analysis. **(C) **ZF domain of PRDM9^Cst^. The letters in the boxes show the amino acids at positions -1, 3, and 6 relative to the α-helix of each finger.

Given the diversity of the PRDM9^Cst ^binding sites, we chose the Hlx1 DNA sequence to further characterize the binding specificities of PRDM9 and to determine the nucleotide specificity of each position in the binding site. Because it was not possible to test all 10^19 ^combinations of base pairs in a 31 bp oligo, we used a competition assay to determine how binding was affected when each base pair was replaced in turn by its three possible alternatives. For this, we first determined the kinetics of replacement of an already bound double-stranded oligo by a competitor (see Additional file 8, Figure S6A). The replacement rate was very slow, indicating very tight binding; reduction of the signal by unlabeled competitor was apparent only after 4 hours of incubation. This made possible a competition assay in which unlabeled mutated oligos were pre-incubated with expressed PRDM9^Cst ^for 1 hour, then one-twentieth the number of molecules of labeled oligo of the original sequence were added, and the mixture was incubated for an additional 4 hours. At the end of the incubation, the extent of binding of the labeled oligo was tested by using the electrophoretic mobility assay (see Additional file 8, Figure S6B). Because the presence of a biotin tag at the end of the minimum binding sequence diminishes binding affinity, the labeled oligo was 75 bp long, with the binding site located in the middle, while the competing oligos were 36 bp long, including two additional base pairs at the 5' end and three additional base pairs at the 3' end relative to the minimal 31 bp binding site. In this assay, the ability of a mutated oligo to reduce subsequent binding by labeled oligo provided a measure of its ability to bind PRDM9; appearance of a strong signal band indicated that the unlabeled mutated oligo failed to bind PRDM9, a weaker signal indicated lower binding strength, and lack of any signal indicated very strong binding. The results of these tests are presented (Figure 5A; see Additional file 8, Figure S6C), and summarized in graphical form (Figure 5B), where we assumed the 5'-3' orientation of the DNA to be relative to the N-C orientation of the ZF array, based on its much better fit to the computer-predicted binding sequence, rather than the reverse.

The 31 positions of the Hlx1 site varied greatly in their specificity. As a simple measure of the degree of specificity, we calculated the standard deviation (SD) of the measured binding affinities at each position (see Additional file 9, Table S3). Using this indicator of specificity, the positions fell into three groups: 13 of low specificity (SD = 0.03 to 0.12), 10 of moderate specificity (SD = 0.16 to 0.36), and 8 with the highest specificity (SD = 0.45 to 0.52), located at fingers 4, 5, and 6 near the center of the binding site. The weakest specificity was at the C-terminal end, fingers 9 to 11, corresponding to positions 25 to 33 of the binding site. Given that these fingers are nevertheless required for binding, it is possible that they function in a non-sequence-specific manner, for example by stabilizing the contact between PRDM9 and DNA through charge interactions involving the phosphate backbone. In this regard, it is interesting that these fingers seem to be less subject to evolutionary selection; the last two fingers are invariant between mouse alleles, and the next has only a single amino acid substitution, N for V. Replacement of nucleotides 1 to 2 and 34 to 36, adjacent to the shortest 31 bp sequence (see Additional file 8, Figure S6C), also affected binding affinity, lending further support to the possibility of more complex interactions between DNA and ZFs.

### Magnesium inhibition

Our data suggesting that binding might involve mechanisms other than interaction with nucleotides in the major groove of DNA, as presently assumed [33], prompted us to test the effect of Mg^2+ ^ions, which are well known to interact with polyphosphates. We found that binding is strongly inhibited at concentrations above 1 mmol/l and nearly completely inhibited by 10 mmol/l (see Additional file 10, Figure S7). This inhibition was essentially identical for both the PRDM9^Dom2 ^and PRDM9^Cst ^variants at all four hotspots tested. The possible involvement of the phosphate backbone of DNA in binding to PRDM9 seems to be a particular characteristic of PRDM9 rather than a general characteristic of ZF proteins, as it is notably different from the effects of Mg^2+ ^on DNA binding by the ZF proteins WT1 and EGR1 [29]' for those proteins, millimolar concentrations of Mg^2+ ^activated binding, and higher concentrations had little inhibitory effect [29].

## Discussion

Taken together, our results confirm that the binding seen with PRDM9 expressed in *E. coli *correctly recapitulates the biological specificities of PRDM9, including its allelic specificity, the haplotype specificity of its target, and the location of the binding sites near the peaks of hotspot H3K4 trimethylation detected *in vivo*. Moreover, the correlation between the binding affinities of different Hlx1 haplotypes and their genetically measured rates of crossover also suggest that the strength of the physical binding of PRDM9 to hotspot sequences may determine the efficiency of recombination initiation. There was little sequence similarity between the several binding sites for the same PRDM9 variant, and the binding specificities of individual fingers appeared to be context-dependent. Mutational analysis of the Hlx1 binding site indicates considerable variation between the ZFs in their contribution to overall binding affinity and the probable involvement of the DNA phosphate backbone.

These findings have relevance to the general role of ZF arrays as a DNA-recognition motif in biology, given that ZF proteins are the most common DNA regulatory protein in vertebrate genomes, comprising over 4% of all protein-coding genes in humans, and that half of these contain 10 or more fingers. They are almost as common in invertebrates, in which they were first described [17]. The issues surrounding the DNA binding specificities of ZFs are both biological and chemical. Biologically, we want to derive a consensus binding sequence whose parameters predict the location and relative binding affinity of genomic binding sites, and then describe how these affinities relate to biological outcomes. Chemically, we want to understand how the specificity of binding sequences is determined by the atomic and molecular interactions between protein and DNA.

PRDM9 provides a particularly useful system for addressing these issues. It is highly variable, with multiple variants available both within and between species. It is a multiply fingered protein, providing the opportunity to examine interactions between repeat fingers in the same protein. In addition, there are many thousands of binding sites in genomes, providing a considerable source of experimental material. To our knowledge, the data we now report provide the first detailed definition of the DNA binding specificities of a ZF protein with a long (> 10) array of contiguous fingers. What has emerged is a picture of considerable complexity, one that raises as many or more questions than it answers.

### Binding-site positions

It was somewhat surprising to find that PRDM9 binding sites are not uniformly positioned in relation to hotspot centers. One possible explanation for this asymmetry could be the presence of crossover refractory zones created by the nature of adjoining DNA sequences that create directionality in the spreading of the Holliday junctions away from initiation sites [30]. However, our genetic data point to a more likely alternative. The sequence of the Pbx1 hotspot is identical in B6xCAST and B6xB6. CAST-1T crosses, but the crossovers in the former cross are resolved on both sides of the binding site, whereas the eight-fold larger number of crossovers in the latter cross are almost exclusively on one side of the binding site. In the absence of possible *cis *effects, this suggests a role for additional *trans*-acting factors in affecting the directional processing of recombination intermediates, with attendant consequences for overall crossover rates at this hotspot.

### Extent of ZF usage

A notable feature of PRDM9 is the required use of all, or nearly all, of its contiguous fingers for binding DNA, as evidenced by the requirement for 35 to 37 bp for PRDM9^Dom2^, which has 12 fingers, and 30 to 33 bp+ for any binding activity by PRDM9^Cst^, which has 11 fingers (the plus indicating that binding is further enhanced at the Hlx1 hotspot by an additional 3 bp, one of which shows considerable nucleotide specificity). This long binding array presents a topological issue, as it seems that at least five to six fingers make base pair-specific contacts with DNA. Moreover, detailed analysis of the Hlx1 binding site revealed that multiple nucleotide positions between positions 5 and 21 show appreciable nucleotide specificity, without any gaps longer than 2 bp. If, as in the case of other ZF proteins, these specific contacts form in the major groove of DNA, it suggests that the ZF domain of PRDM9 can bind continuously along the major groove for more than one turn of the DNA double helix. In the context of intact cellular DNA, this would require a snake-like winding of the DNA around its target, which is in marked contrast to the accepted rule that ZF proteins do not use more than three contiguous fingers to bind DNA [22], and thus requires further explication. Given the further observation that binding is inhibited by magnesium, which complexes with polyphosphates, the requirement for additional fingers with low sequence specificity may indicate the importance of non-specific charge interactions between negatively charged phosphate groups and the positively charged Zn^2+ ^and histidine residues.

### Binding-site relatedness

The three DNA binding sites identified for the PRDM9^Cst ^variant bear little ostensible resemblance to each other, with little identity between the nucleotide positions along the entire length of these sequences. However, a more subtle relationship between the three sequences can be detected when they are compared with the mutation-analysis data for Hlx1. This suggests that both the strongest binding within an Hlx1 site and most of the sequence matches between the three binding sites occur over the nucleotides binding to the first six fingers of the PRDM9^Cst ^ZF array. The binding rules are obviously complex, and this complexity was emphasized when we examined the Hlx1 hotspot in detail; the mutation analysis showed variable stringency along its length for base-pair identities. At every position, two, three, and often all four bases could serve, albeit not always equally well.

### Context dependence of ZF specificity

In addition to the weak similarity between binding sequences, there was also a strong context dependence of DNA binding, with the binding properties of a finger being dependent on its location in the array. Although the second, fifth, seventh, and ninth ZFs of PRDM9^Cst ^have identical amino acid sequences, they did not bind related trinucleotide sequences either within or between hotspots (see Additional file 5, Figure S5).

### Evolutionary context

Previous three-dimensional studies of ZF DNA interactions suggest a model in which four amino acids in each finger (positions -1, +2, +3, and +6) bind four consecutive base pairs in DNA. The last of the four bases shares its binding with the next finger, giving a repeating pattern of 3 bp per finger (plus 1 bp for the total possible length of the binding site) [25]. Computer analyses of multiple 3D structures indicate that all four amino acids are equally important [22]; however, the evolutionary data suggest otherwise for PRDM9. The amino acid composition of its ZFs undergoes rapid evolutionary selection to generate new hotspots as existing hotspots undergo continual loss by mutation [31]. Notably, position +2 is essentially invariant in the face of extraordinarily high replacement rates at positions -1, +3, and +6 [8,10,32,33], suggesting that, at least for PRDM9, this amino acid plays a lesser role in determining DNA binding specificity.

## Conclusions

Our findings introduce considerable complexity into efforts to understand DNA binding by long-array ZF proteins such as PRDM9. The binding sites for the PRDM9^Cst ^variant are only very subtly related; identical ZFs show little constancy in the trinucleotides they bind, and a position weight matrix approach [27] failed in a search for a DNA binding motif common to genetically identified hotspots on mouse chromosomes 1 and 11 [5,28]. Perhaps most surprising is the finding that binding requires DNA sequences involving more than one helical turn, even though the terminal nucleotide positions show little sequence specificity. To our knowledge, PRDM9 is the first long-array ZF protein to have its DNA binding specificity at separate binding sites compared in detail. At issue now are the biological question of how to solve the binding rules for PRDM9 and the chemical question of whether the binding complexities of PRDM9 are shared by the hundreds of other long-array ZF proteins that carry out a great diversity of biological functions.

## Materials and methods

### Cloning and expression of mouse *Prdm9*

Full-length *Prdm9 *was amplified from cDNA prepared from the testes of CAST/EiJ (CAST) and C57BL/6J (B6) mouse strains using primers Prdm9F-HisB and Prdm9R-HisB (for sequences of primers used for cloning and sequencing, see Additional file 11) and cloned into pBAD/HisB (Invitrogen Corp., Carlsbad, CA, USA) using *Xho*I and *Hin*dIII restriction sites added to the 5'-ends of the primers. The identity of the cloned products was confirmed by sequencing (see Additional file 12). The plasmids were transformed into TOP10 *E. coli *cells, allowing arabinose induction of protein synthesis. For production of PRDM9 protein, the cells were grown overnight in Luria broth and then incubated an additional 4 hours with the addition of 0.02% arabinose. Total cell lysate was prepared in native binding buffer (50 mmol/l sodium phosphate pH 8 and 500 mmol/l NaCl) in accordance with the manufacturer's specifications, and stored at -80°C until use, when it was heated at 50°C for 15 minutes to inactivate bacterial DNAses. The production of full-length protein was confirmed by western blotting using an antibody to the N-terminal his tag. Crude bacterial lysates used in the *in vitro *binding assays contained 15 to 30 μg/ml expressed PRDM9 as estimated by ELISA.

### Histone methylation assay

Crude bacterial extract containing induced/non-induced PRDM9 variants or empty pBAD/HisB vector were heated for 15 minutes at 50°C, then used for a histone methyltransferase assay. The reaction mixture contained 20 μl 2× HMT buffer (50 mmol/l Tris-HCl pH 8 and 20% glycerol), 2 μl S-adenosinmethionine (100 μg/ul; Sigma-Aldrich, St Louis, MO, USA), 10 μl bacterial extract, 0.2 μl histone substrate (0.5 μg/μl recombinant H3K4-me2; Active Motif, Carlsbad, CA, USA), and 7.8 μl water. The reaction was kept at 34°C for 30 minutes.

Several commercially available antibodies were used to detect specific H3K4me3 signals. All tested antibodies showed cross-reactivity with H3K4me2. Therefore, we estimated the level of H3K4 trimethylation by induced PRDM9 relative to controls with induced empty vector. The results presented here were obtained using the antibody that showed the most consistent discrimination between H3K4me2 and H3K4me3 signals (recombinant H3K4-me2; Active Motif).

Histone methyltransferase activity was measured by dot blotting. The reaction mixtures were blotted onto nitrocellulose membrane and the trimethylated H3K4 was detected with anti-H3K4me3 antibodies (Active Motif). The chemiluminiscent signal was visualized (Lumiglo Kit; Cell Signalling Technology, Danvers, MA, USA) and scanned with an image scanner (ImageQuant.LAS-4000; GE Healthcare, Princeton, NJ, USA) The fraction of the specific H3K4me3 signal was calculated as

$${\text{F}}_{\text{H3K4me3}}=\left({\text{S}}_{\text{ind}}-{\text{S}}_{\text{un}}\right)/)\left({\text{S}}_{\text{H3K4me3}}-{\text{S}}_{\text{H3K4me2}}\right),$$

where F_H3K4me3 _is the fraction of the specific signal, S_ind _and S_un _are the signals of induced and uninduced extracts, and S_H3K4me3 _and S_H3K4me2 _are the signals of commercial H3K4me3 and H3K4me2, respectively.

### Electrophoretic mobility shift assay

Using an electrophoretic mobility shift assay (EMSA) (LightShift Chemiluminescent EMSA Kit; Pierce Biotechnology ThermoScientific, Rockford, IL, USA), a biotin-labeled test oligo and PRDM9 were allowed to bind in a mixture containing 10 mmol/l Tris (pH 7.5), 50 mmol/l KCl, 1 mmol/l dithiothreitol, 50 ng/μl poly(dI/dC), 0.05% NP-40, 0.5 pmol/l of labeled double-stranded oligo, and 3 μl of bacterial extract in a total volume of 20 μl. When necessary, 10 pmol/l of cold competitor or non-competitor was added. The reaction mixture was incubated for 40 minutes and separated on 5% PAA gel in 0.5× Tris-borate-EDTA (TBE) buffer, which was pre-electrophoresed for 30 minutes. After electrophoresis, the separated products were transferred onto nylon membrane by wet transfer in 0.5× TBE at 380 mA for 1 hour. The membrane was cross-linked by UV light at 120 mJ/cm^2 ^(UV Stratalinker 2400; Agilent Technologies, Santa Clara, CA, USA). Biotin-labeled products were detected by the chemiluminescence signal produced by the binding to streptavidin-peroxidase conjugate in accordance with the manufacturer's specification, and the chemiluminesce signal was recorded (G:BOX system; Syngene, Frederick, MD, USA).

Single-stranded oligos were biotin-labeled at the 3'-end *Biotin 3' End DNA Labeling Kit; Pierce Biotechnology ThermoScientific) in accordance with the manufacturer's specifications. Complementary oligos were then mixed, denatured at 95°C for 2 minutes, and annealed by cooling down to room temperature in a water bath. When a PCR product was labeled, the two strands were separated by heating at 95°C for 5 minutes. The tube was immediately transferred to ice, and then the product was labeled and re-annealed as above.

The bands were quantified using Gene Tools software (Syngene, Frederick, MD USA), using the 'Manual bands' and 'Absorption' settings. Equal-sized rectangles were drawn around each shifted and unshifted band present, and the absorption density was scored automatically by the software. The proportion of shifted band was calculated as S/(S+U), where S is the density of the shifted band and U is the density of the unshifted band.

The DNA binding sites of hotspots were localized by testing whether the electrophoretic mobility of biotin end-labeled DNA sequences was altered after binding to the PRDM9^Cst ^and PRDM9^Dom2 ^proteins expressed in *E. coli*. All binding experiments were performed using crude *E. coli *extracts, because every attempt to purify PRDM9 resulted in insolubility and loss of activity. The specificity of binding was confirmed by appropriate controls, including induced cells that had been transformed with empty vector, and the host cells alone. Additionally, the variant-specific binding of PRDM9 to hotspot sequences was confirmed by isolation of protein-biotinylated DNA complexes onto streptavidin beads and detection of PRDM9 by western blotting with antibodies against its C-terminal end (see Additional file 13, Figure S8).

To localize binding sites, a tiling approach was used, in which PCR-amplified fragments of 200 to 400 bp, overlapping by 50 bp, were tested for their ability to bind PRDM9 variants (Figures 1A, 2A, 3A, D; also see Additional file 2, Figure S2B; Additional file 2, Figure S3A; Additional file 2, Figure S4A). Positive fragments were then reduced to less than 100 bp using the same strategy. Because biotin labeling close to the end of a binding-site sequence influences binding, the limiting sequence for each binding site was then determined by comparing the binding ability of progressively shorter, unlabeled oligos against the binding ability of longer, labeled oligos.

### Mutational analysis

For the competition assays testing how single-nucleotide substitutions affect DNA-PRDM9 binding, bacterial extract was incubated with 10 pmol/l of unlabeled double-stranded oligo for 1 hour (all other components except labeled oligo were as given above), then 0.5 pmol/l labeled oligo was added, and the extract was incubated for an additional 4 hours (see Additional file 8, Figure S6A). Under these conditions, appearance of a strong, shifted, labeled DNA band indicates that the mutated sequence shows weak or no binding, whereas a weakly shifted band indicates strong binding by the mutated oligo (see Additional file 5, Figure S5B). The separation and detection conditions were the same as described above. The strength of binding of each mutated oligo to PRDM9^Cst ^was then determined by comparing its ability to replace previously bound, labeled, Hlx1 oligo with the abilities of the unlabeled oligo and an unlabeled oligo with a randomly scrambled Hlx1 sequence. Competition was calculated as:

$${\text{B}}_{\text{mut}}=\left({\text{S}}_{\text{un}}-{\text{S}}_{\text{mut}}\right)/)\left({\text{S}}_{\text{scr}}-{\text{S}}_{\text{un}}\right),$$

where B_mut _is the relative change in binding strength of the mutated oligo, and S_un_, S_mut_, and S_scr _are the proportions of shifted bands of the unmutated, mutated, and scrambled oligos, respectively.

### Streptavidin pull-down and western blotting

Protein-biotinylated DNA complexes were isolated on streptavidin beads. PRDM9 variants were expressed in *E. coli *and bound to biotinylated oligos representing binding sites, as described above for the EMSA experiments. The complexes were then isolated on T1 streptavidin-coated beads (Dynabeads; Invitrogen Dynal, Oslo, Norway) and washed three times with EMSA binding buffer, then the bound proteins were released from the beads by adding 0.1% SDS. The proteins were separated on 4 to 20% SDS gels and transferred onto polyvinylidene difluoride (PVDF) membrane, and the specificity of PRDM9 binding was detected on western blots using mouse antibodies against the C-terminal part of PRDM9.

### Chromatin immunoprecipitation for H3K4-me3

Crude isolation of spermatocytes from B6 and B6xCAST F1 juvenile mice was performed as reported previously [34] with minor modifications.

Spermatocytes were isolated from testes of 12-dpp juvenile mice. Cross-linking of spermatocytes was performed by addition of formaldehyde to a final concentration of 1% and incubation at room temperature for 10 minutes with constant rotation. Cross-linking was stopped by dropwise addition of glycine to a final concentration of 125 mmol/l, followed by incubation with rotation for 5 minutes at room temperature. Cells were washed twice, separated by centrifugation at 2000 *g *for 5 minutes at 4°C, and resuspended in 1 ml PBS. After the final wash, hypotonic lysis buffer (10 mmol/l Tris-HCL pH 8.0, 1 mmol/l KCl, 1.5 mmol/l MgC_l2_) was added at a concentration of 5 × 10^6 ^cells/ml supplemented with 1 mmol/l phenylmethanesulfonylfluoride (PMSF) and 1× protease inhibitor cocktail (PIC; Sigma-Aldrich) and incubated for 30 minutes at 4°C with rotation to shear the cellular membrane. Nuclei were pelleted by centrifugation at 10,000 *g *for 10 minutes and resuspended in MNase buffer (50 mmol/l Tris, 1 mmol/l CaCl, 4 mmol/l MgCl, 4% NP-40) at 26 μl/10^6 ^cells, supplemented with 1 mmol/l PMSF and 1× PIC. Chromatin was fragmented and solubilized by addition of MNase (USB, Cleveland, OH, USA) at 15 U per 5 × 10^6 ^cells, followed by incubation for 2 minutes at 37°C. Nuclease activity was stopped by addition of EDTA to a final concentration of 10 mmol/l and incubation at 4°C for 5 minutes. Soluble chromatin was clarified by centrifugation at 4°C for 10 minutes at top speed, then the supernatant was transferred to a clean tube and the centrifugation repeated. H3K4me3 antibody (Millipore Corp., Billerica, MA, USA) was prebound to 20 μl protein-G beads (Dynabeads; Invitrogen Corp.) following the manufacturer's protocol. Antibody-bound beads were washed twice with 100 μl of immunoprecipitation (IP) buffer (RIPA 50 mmol/l Tris pH 8.0, 150 mmol/l NaCl, 1.0% NP-40, 0.5% Na deoxycholate, 0.1% SDS supplemented with 50 mg/ml BSA, and 0.5 mg/ml salmon-sperm DNA) and resuspended in a final volume of 75 μl IP buffer with PMSF and 1× PIC. Undiluted chromatin (25 μl; around 10^6 ^cell equivalents) was added to the beads, and incubated with rotation at 4°C for 2 hours. The chromatin-bound beads were washed three times with 100 μl IP buffer and then twice with 100 μl TE buffer pH 8.0 before elution with 125 μl elution buffer (1% SDS, 20 mmol/l Tris-HCL pH 8.0, 200 mmol/l NaCl, 5 mmol/l EDTA) supplemented with 50 μg/ml Proteinase K (Sigma-Aldrich). Elution was carried out by incubation at 68°C for 2 hours with vigorous shaking at top speed in a thermal mixer (Thermomixer; Eppendorf, Westbury, NY, USA) to reverse cross-links and digest all proteins. An equal aliquot (25 μl of 'input' chromatin was diluted to a final volume of 125 μl with elution buffer and handled in parallel. DNA was recovered from the beads using magnetic separation, placed into a clean tube, and then purified using commercially available methods following the manufacturer's protocol for PCR clean up (Qiagen Inc., Valencia, CA, USA). Purified DNA was eluted in a total volume of 200 μl 10 mmol/l Tris pH 8.0.

Primers were designed using OligoPerfect™ primer design software (Life Technologies, Corp., Carlsbad, CA, USA) using the following parameters, 40 to 60% GC, 57 to 63°C Tm, with a product size of 8 to 120 bp. Each qPCR reaction was performed in triplicate 20-μl reactions using a commercial kit (Quantifast SYBR Green PCR Kit; Qiagen Inc.) and following the manufacturer's protocol,, then run on a real-time PCR system) (MasterCycler^® ^ep realplex; Eppendorf) for 40 cycles, followed by a melting-curve analysis. Ct values were calculated using an automated threshold and averaged for triplicate experiments. If a PCR reaction failed to amplify in the IgG control, the Ct value for that reaction was arbitrarily set to 35 cycles, typically around 12 cycles less than the input sample (or less than 0.024%). Reactions for both the input and ChIP sample were seeded with 2 μl of DNA. The percentage of chromatin-bound (fraction bound) was calculated by:

$$2\left(\text{C}{\text{t}}_{\text{Input}}-\text{C}{\text{t}}_{\text{ChIP}}\right)\times 100.$$

### Bioinformatic search for a DNA binding motif

A position weight matrix (PWM) was created (Table S2) based on the mutational analysis of the Hlx1 binding site, and a matrix matching algorithm from the software package Motif Occurrence Detection Suite [27] was used. The weights obtained for the matrix were optimized by maximizing the score for the detection of the Hlx1 binding site within a scan against the entire Hlx1 hotspot region. Subsequently, the PWM was used in a scan against all hotspots less than 10 Kb in size located on chromosome 1, along with control regions adjacently positioned before and after each hotspot; each control region was sized equivalently to its corresponding hotspot. To minimize the effects of multiple testing, only matches with *P*-values below 10^-5 ^were considered.

## Abbreviations

B6: C57BL/6J mouse strain; BSA: bovine serum albumin; CAST: CAST/EiJ mouse strain; ChIP: chromatin immunoprecipitation; DSB: double-strand breaks; ELISA: Enzyme-linked immunosorbent assay; EMSA: electrophoretic mobility shift assay; H3K4: histone 3, lysine 4; H3K4-me3: trimethylated H3K4; IP: immunoprecipitation; NCBI: National Center for Biotechnology Information; PBS: phosphate-buffered saline; PRDM9: PR domain member 9; PMSF: phenylmethanesulfonylfluoride; PVDF: polyvinylidene difluoride; PWM: position weight matrix; SDS: sodium dodecyl sulfate; SNP: single-nucleotide polymorphism; TE: Tris-ethylenediaminetetraacetic acid; TBE: Tris-borate-ethylenediaminetetraacetic

## Competing interests

The authors declare no competing interests.

## Authors' contributions

PMP and KP designed the experiments; TB, EDP, and CEB performed the experiments; MW performed the bioinformatical analyses; and PMP and KP wrote the paper. All authors read and approved the final manuscript.

## Supplementary Material

Additional file 1 **Figure S1. *Escherichia coli*-expressed PRDM9 protein variants retain their H3K4 trimethylation activity**. The Additional material contains maps of all hotspots studied in this paper, their sequences, additional figures and tables highlighting specific points in the paper, and the sequences of the oligos used for mapping. Click here for file

Additional file 2 **Figure S2. The PRDM9^Dom2 ^binding site of Pbx1**. The Additional material contains maps of all hotspots studied in this paper, their sequences, additional figures and tables highlighting specific points in the paper, and the sequences of the oligos used for mapping. Click here for file

Additional file 3 **Figure S3. The PRDM9^Cst ^binding site of Hlx1**. The Additional material contains maps of all hotspots studied in this paper, their sequences, additional figures and tables highlighting specific points in the paper, and the sequences of the oligos used for mapping. Click here for file

Additional file 4 **Figure S4. The PRDM9^Cst ^binding site of Esrrg-1**. The Additional material contains maps of all hotspots studied in this paper, their sequences, additional figures and tables highlighting specific points in the paper, and the sequences of the oligos used for mapping. Click here for file

Additional file 5 **Figure S5. Alignment of the three PRDM9^Cst ^binding sites**. The Additional material contains maps of all hotspots studied in this paper, their sequences, additional figures and tables highlighting specific points in the paper, and the sequences of the oligos used for mapping. Click here for file

Additional file 6 **Table S1. Persikov algorithm predictions for 200 bp regions surrounding binding sites**. The Additional material contains maps of all hotspots studied in this paper, their sequences, additional figures and tables highlighting specific points in the paper, and the sequences of the oligos used for mapping. Click here for file

Additional file 7 **Table S2. Position-weighted matrix based on the results of the electrophoretic mobility shift assay (EMSA)**. The Additional material contains maps of all hotspots studied in this paper, their sequences, additional figures and tables highlighting specific points in the paper, and the sequences of the oligos used for mapping. Click here for file

Additional file 8 **Figure S6. Nucleotide substitution analysis of Hlx1**. The Additional material contains maps of all hotspots studied in this paper, their sequences, additional figures and tables highlighting specific points in the paper, and the sequences of the oligos used for mapping. Click here for file

Additional file 9 **Table S3. Binding changes at each position along the Hlx1 binding site caused by a mutated nucleotide**. The Additional material contains maps of all hotspots studied in this paper, their sequences, additional figures and tables highlighting specific points in the paper, and the sequences of the oligos used for mapping. Click here for file

Additional file 10 **Figure S7. Effect of Mg^2+ ^on PRDM9-DNA binding**. The Additional material contains maps of all hotspots studied in this paper, their sequences, additional figures and tables highlighting specific points in the paper, and the sequences of the oligos used for mapping. Click here for file

Additional file 11 **Additional experimental procedures: oligos used for analysis of binding**. The Additional material contains maps of all hotspots studied in this paper, their sequences, additional figures and tables highlighting specific points in the paper, and the sequences of the oligos used for mapping. Click here for file

Additional file 12 **Sequences of Prdm9^Dom2 ^and Prdm9^Cst ^cDNA cloned in pBAD/HisB**. The Additional material contains maps of all hotspots studied in this paper, their sequences, additional figures and tables highlighting specific points in the paper, and the sequences of the oligos used for mapping. Click here for file

Additional file 13 **Figure S8. PRDM9 binds to its targets in an allele-specific manner**. The Additional material contains maps of all hotspots studied in this paper, their sequences, additional figures and tables highlighting specific points in the paper, and the sequences of the oligos used for mapping. Click here for file
