# Supplementary material for: DNA binding specificities of the long zinc-finger recombination protein PRDM9
Source: Genome Biol. 2013 Apr 24;14(4):R35. doi: 10.1186/gb-2013-14-4-r35 (PMC4053984; doi:10.1186/gb-2013-14-4-r35)

Additional file 1:

Figure S1. *E.coli*-expressed PRDM9 variants retain their H3K4-trimethylation activity. Total bacterial extracts of induced or uninduced *Prdm9* were incubated with total histones and S-adenosil-methionine, dot-blotted, and developed with anti-H3K4me3 antibodies. Upper row, uninduced extracts; lower row, induced extracts. Commercial preparations of H3K4me2 and H3K4me3 were used as controls for the antibody specificity. The numbers below show the fraction of H3K4me3-specific signal relative to the commercial H3K4me2 and H3K4me3 controls.

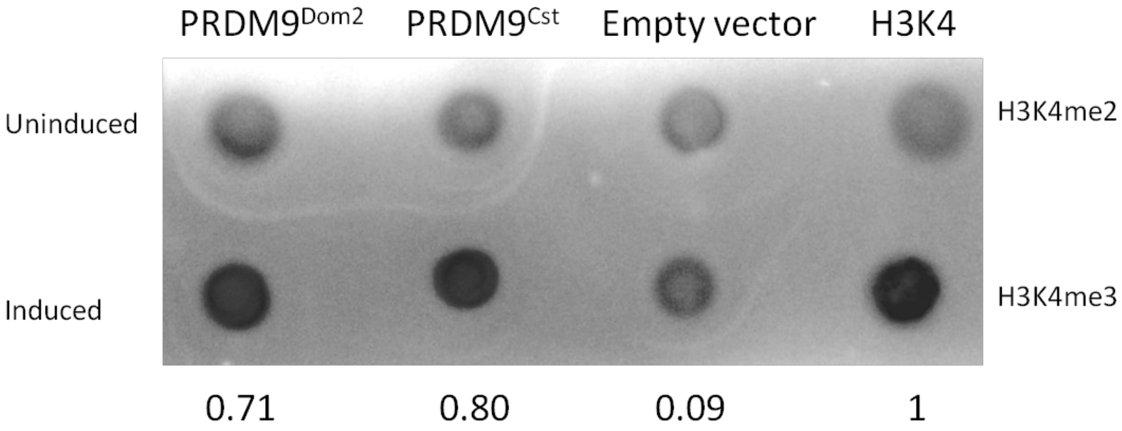

Supplement: Additional file 1 — Figure S1. Escherichia coli-expressed PRDM9 protein variants retain their H3K4 trimethylation activity. The Additional material contains maps of all hotspots studied in this paper, their sequences, additional figures and tables highlighting specific points in the paper, and the sequences of the oligos used for mapping. [file gb-2013-14-4-r35-S1.PDF]
