# Supplementary material for: DNA binding specificities of the long zinc-finger recombination protein PRDM9
Source: Genome Biol. 2013 Apr 24;14(4):R35. doi: 10.1186/gb-2013-14-4-r35 (PMC4053984; doi:10.1186/gb-2013-14-4-r35)
Supplement: Additional file 2 — Figure S2. The PRDM9Dom2 binding site of Pbx1. The Additional material contains maps of all hotspots studied in this paper, their sequences, additional figures and tables highlighting specific points in the paper, and the sequences of the oligos used for mapping. [file gb-2013-14-4-r35-S2.PDF]

Additional file 2:

Figure S2. The $PRDM9^{Dom2}$ binding site of Pbx1.

(A) **Activity of Pbx1 in different mouse crosses.** For better visualization, recombination activities in crosses BxB.C-1T and WxC are shown above the horizontal axis, and activities in crosses BxC, BxP, and WxP are shown below the horizontal axis. (See the table for full strain names and associated *Prdm9* alleles.) The hotspot is active only in crosses involving C57BL/6J as one of the parental strains. Crosses WxC and WxP, which lack recombination activity in this region, are plotted slightly above or below the horizontal axis so that the lines can be seen. The position of the tiled region shown in Figure 1B is outlined with a double-headed arrow.

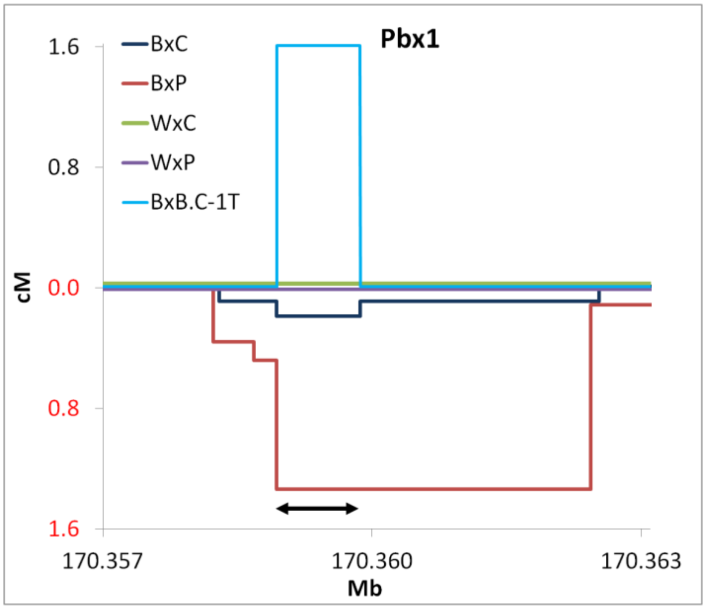

Strain abbreviation	Strain name (*Prdm9* allele)
B	C57BL/6J ($Prdm9^{Dom2}$)
C	CAST/EiJ ($Prdm9^{Cst}$)
P	PWD/PhJ ($Prdm9^{Msc}$)
W	WSB/EiJ ($Prdm9^{Dom3}$)
B.C-1T	Congenic strain carrying 100 Mb of a CAST sequence on Chr 1 on a C57BL/6J background ($Prdm9^{Dom2}$)

Abbreviations as defined in Parvanov ED, Petkov PM, Paigen K: **Prdm9 controls activation of mammalian recombination hotspots.** *Science* 2010 **327**:835.

(B) Binding of PCR-amplified fragments tiling Pbx1 to PRDM9^{Dom2}. Lanes 1–5: tiling labeled fragments 1–5 incubated with crude bacterial extract containing PDRM9^{Dom2}. Only fragment 1 shows specific band shift. The sizes of the tiling fragments (lowest bands) are 204, 193, 196, 187, and 187 bp, respectively. The two bands above are non-specific and probably caused by the presence of biotin-containing proteins in crude bacterial extract.

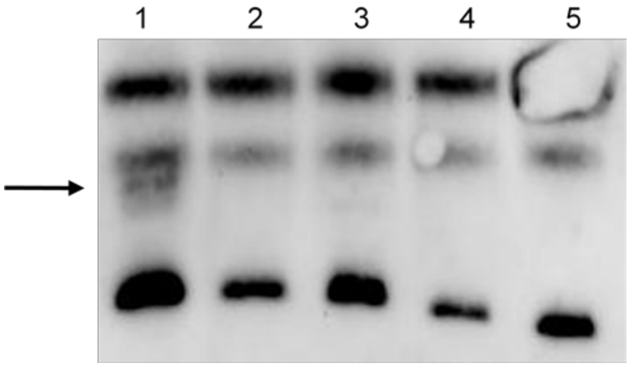

(C) Fine mapping of Pbx1 by direct binding of biotin-labeled double-stranded oligos. The numbers below each lane show the size of labeled oligos; the shifted band is indicated by the arrow. The sequences of the oligos used are shown in Additional experimental procedures in Additional file 12. The non-specific bands in all lanes are probably caused by the presence of biotin-containing proteins in crude bacterial extract.

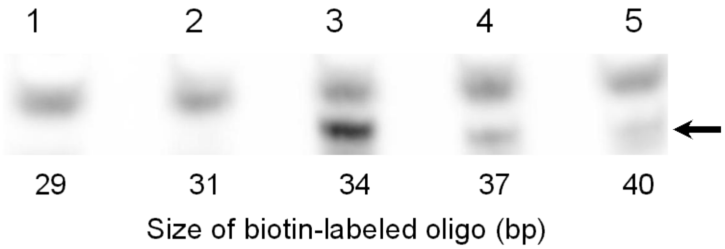

(D) Sequence of Pbx1. The flanking SNPs are in bold and the PRDM9 [Dom2] binding site is in bold, underlined. Positions of flanking SNPs:

- rs31416165 [T/A]: 170, 451, 754
- rs31415340 [T/G]: 170, 452, 394
- rs30796712 [C/G]: 170, 453, 324 (NCBI B37)

GTAAATTTTGG**[T/A]**GATTTGGATAGGAAGTATCCTTCCAAGGCTTTTGAGACCTTGGGGATAGCTTTA
GCTCCCCCATGTCTCTTTTCCTCTTGTCTATTAGGAGGTGAGTGGGTTCCAGGACACACTCCCCTACCAT
GATTCATTCTCTCTCTCTCTCTCTCTCTCTCTCTCTCTCTCTCTCTCTCTCTCTCTCTCTCTCTCTCTCT
CTCTCACCATAGATTTAGAAACATACGGAGTTGACCATCCTGGACTGAATCCTCTGAAGGAACAAGTCAA
ATGAAATACTTCTTCCCTTACATTGTTTATGTCAGGAATTTTAGTCACAGCTTTGCAAAAGTAACTAACA
ATTCAAACTTTAAATCATCGGGGCTTCCACATGCACATAGGCTTGCTTGGGCCATTTTTATATCTAGAAT
CTTGCTGTATATAGCTGATACAGCTGGCTTGCTTGGTGCCTTCAGTTTTTCTTGGTAGACATCTCCTACC
TTTGATATTTTCAGTATTATGGGGAAGGGGGGTGTCTCTATTTCTGGTTAGGTTTTTTCATCATAGATTC
ATGAATCACCTATTCAAGGAAGGGACTGTTACAGACTCTAAACCTGTCAAAGGCTTTGAAGACTTTTTTT
TTTTCTGAAATCTGAGCAGATACTA**[T/G]**CATATG**GAGAACTTACAAAGGTAGGACGTAAATGGAG**
TGTTTACACCACCGTACTAGAGGAAGTCAAAGTGTGTTCTCATTTTGAAACAGGTTAGACATTTCTTGTA
AACACAAACTTACCATGTAATCTATAAATTCCTATCACAAAAGATTATTCTATAAAAAAGATAGAAACTG
ACATATGAAGGCTCATTTATGAGTCTAAAGTTGTATTATGTCCAGTTTAAGGGAGCAAAAGCAAATGAGG
ATCTCTTTTGGTCCCAGGTACAGGAAAAGATAGAGAGGGCCAATACAATCTATAATGACAAAAGACATAT
TCATAGTTACCTGTGTCAGAGATACAGGGACTTCCTGGGAATGGATACAAGGCCTTTGAGGATGACTGGA
GTATGATTTATTGGTAAGTGTTTTATCTTGATGTGATTGCTGTAGTGTCAATTGGTCCATACTTGTCCAA
AGTATGGACTTGTCGAAGTCCACACTTAAAGTGGATGTATTTTGTATGCGTTACACCTAAATAAACTTTA
CCTTAAATCCAAGTATGAGAAAACTAGAATGATAAAGATAGAGACTGGTAAGAAAGGTAGTGCTTTTCAG
CCTTTTGGTTAAGAGCAAAGCTAGCAAAAGCCAGGGTGGACAATATAAACATTACAATTAACTTTATAAC
AGAGAATTTTGACATTGACCAGCATTCATGCTAAGTGGGAAGTATCTTGTATAACTTTATCTTGCTTCTC
ACTTTGGTTTCTGTTTCTGTTGTTGTTGTGACTTCAGCAGATGAATTCATCAGGACAAAAGAAAATCACG
GCCTTCTGTTTGTAGTGGGGGATGAGCCGGCATGCTTAGTGCACAAGTCATTGAGAAGAATAAGCACCAT
AGGCCCTAGTGACACCGAGCAGCGAGGAACCTACTCTGATATTGTCTTTCTC**[C/G]**GAATCTCATTGGT
CACAGAGCACCTTGTGAAGTTGTTTCCCCTCCTC
