# Supplementary material for: DNA binding specificities of the long zinc-finger recombination protein PRDM9
Source: Genome Biol. 2013 Apr 24;14(4):R35. doi: 10.1186/gb-2013-14-4-r35 (PMC4053984; doi:10.1186/gb-2013-14-4-r35)
Supplement: Additional file 3 — Figure S3. The PRDM9Cst binding site of Hlx1. The Additional material contains maps of all hotspots studied in this paper, their sequences, additional figures and tables highlighting specific points in the paper, and the sequences of the oligos used for mapping. [file gb-2013-14-4-r35-S3.PDF]

Additional file 3:

Figure S3. The PRDM9^{Dom2} binding site of Hlx1.

(A) Binding of PCR-amplified fragments tiling Hlx1 to PRDM9^{Cst}.

Upper panel. Odd numbered lanes (1, 3, 5, 7, 9, 11, 13, and 15) were loaded with tiling labeled fragments 1–8 alone, respectively. Even numbered lanes (2, 4, 6, 8, 10, 12, 14, and 16) were loaded with tiling fragments incubated with crude bacterial extract containing PRDM9^{Cst}. The substantial difference in fragment size is dictated by the presence of several repeated regions at the hotspot. Non-specific bands present in all lanes incubated with PRDM9^{Cst} are probably caused by the presence of biotin-containing proteins in crude bacterial extract or by non-specific binding of biotinylated PCR products to bacterial proteins. Only the band in lane 12 (fragment 6), indicated by the red asterisk, was confirmed as specific by competition assay (lower panel).

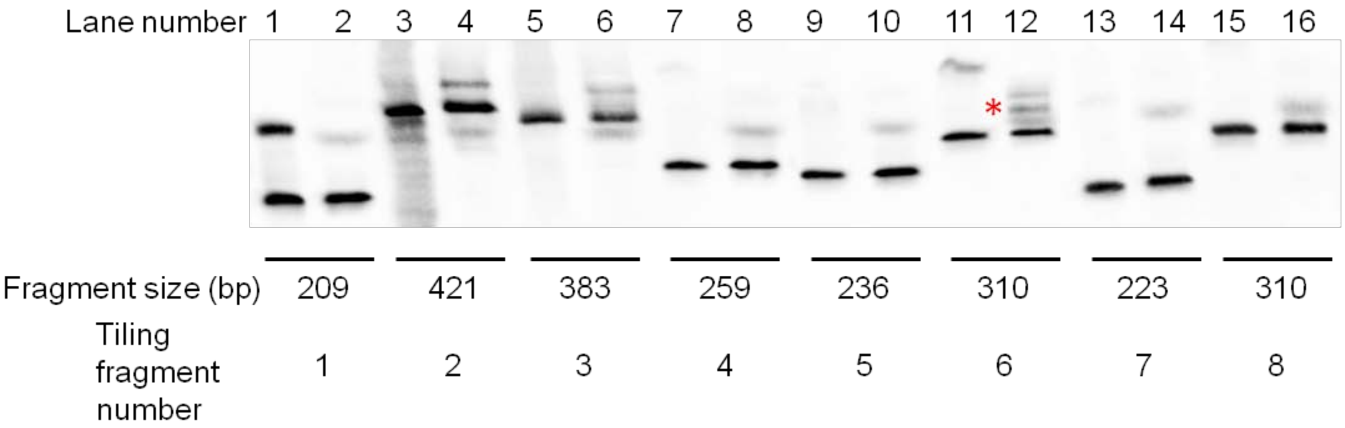

Lower panel. Competition assay for fragment 6. Lane 1, labeled fragment 6; lane 2, labeled fragment 6 + PRDM9^{Cst}; lane 3, labeled fragment 6 + PRDM9^{Cst} + excess of unlabeled fragment 6; lane 3, labeled fragment 6 + PRDM9^{Cst} + excess of unlabeled fragment 8; Unlabeled fragment 6 competes with the labeled fragment whereas fragment 8 does not.

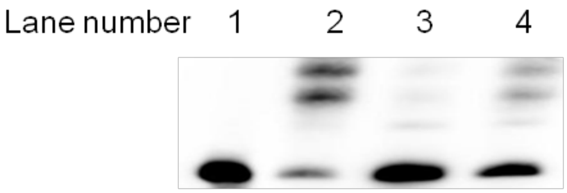

(B) Sequence of Hlx1 with the flanking SNPs in bold and the PRDM9[Cst] binding site in bold, underlined. SNPs between C57BL/6J and CAST/EiJ are shown.

AGAACTGACAGCAAT**[A/G]**GGGCCCAGGTTTGGCTCCAGGAAGCCTTCTGAATTG[C/A]TCTGCTTTC
TCTTAGCTTGCTGCTATCCCAGAACCA[T/G]CACAGACGCCACTCTGCCGGATACAGGCCACATGGAGC
TAAGGCAAGCCATCCCGAACCAGCTACGAGGGAGTCCAGGCAGAAGTGAGCCTAGCCAAGAACGGCGGAG
CCTGGCTCAGCTGACCTCAGCAGAACCGTTCAGCGAGCACAGACTCTTGTT[C/A]CTCCACAACAGCTA
CCTACTAAAGCCGGGAGGCCAGTGGTCCTTGGCCTGTTTCAGGCTCC[G/A]TTTTCATATCTCACCTTA
AAATTCATTCCCTACAATGGAAGAC[-/A]AAAAACTTAGTTATATTATAATTCATCCCTAGCAAATACT
CACTTCCCCTGTCTCT[-/TACTTCTAA]ACATTTTTGATGAACCATGCTTGGTTAAATTAATG[T/C]
TCGATGCTTGTGGGGCTGGAGAGATGACTCAGAGGCTAAGAGCTCACGATGCTCTTCCAGAGGACCCCGG
TTTCAGTTTCCAGAG[C/T]CCACACCAAGTGGCTCACAACTGCCTGTAACTCCAGCTCCAGAGAATTGC
TCTGACCGCCTCAG[G/A]CACCTGCATATACGTGGAACACCTAACATAGACAAAAATAAAAATGCAAGT
AAGTATTTTT[T/A]AAAATACAGTGATTATACCCTTGTTATATAAACGTCACTTATAGATGACTTAT[G
/A]TAATTT[C/A][A/T]AATTGTA[T/A]TTTTTAATTATTTCTTTTTTAATTATCATACAAAGTAAC
ATGTTTCCTTGA[A/G]ATGTTTTCAAATAGCCTTTGTGTTGAAACCCTTAAGTTCCTCTCCATCATCTC
CCTACTCCCTCTCCCAGCTTGTCCCCTCTACCTTCAGTATTTTCTTTCTCTTTTTCATGTCACATGTGAA
GGGACATAGGGAAGGGGAATTGTATTATGAAAGAGAGGCCACCAAGACTCAAGACCCCTGGAAGTACTTT
AAGACACAGAAGTCTTCAAGCAAGCTGTGACCTGAGACATTGGCTGCTCTTTAAAACAGTGGAAGTCGTC
ATTGAAACACCATTTAGGTAATGGAGACACAAATAGAAACAACTACCAAGAACTTCCCATCAGCAGTGAA
AGGGTCACTTCCTTTTTTTTTTTTTTTTTTTTTTTTGGTTAAATGCCCACCCTGAACACCACCACTCCTT
TCCAGGCAGAATTCTGTCGATAACCCATTCCAAGCAAGACTCTTGAACTTCCACAGCCCTAGGGTTTTCT
TTGGAGGGAAAGTATAAGGATGTTTTGTCCTACCTCGGGTTGCTGGCTTCTTAAATTGATCCTTGCTACC
CTCCCCCTGCTCATTAGCCTTGCATGTCCTGACCTACTCTCTTCTTTCTTTCCTTTTTCCTCTATTTCCC
CAAATCATTACCCTTACATACCTAAAAACTGCACTAGT[T/C]CCTAGCCCTTCCTTATCACAGCATACA
TTCCTTACTGTTTGGGTATAAGGTATCCAACCACCCAGATCATCTTTGCTTGAAATACTAACTACATAGA
GCTGTAAGCTTCAACAGTATCAAAGCCTGACCCTCATTTGGCAGGGCTCCAGAGAAGCAGGGACACTGTC
AGGGAAACTGGTTTGCACCTTACTTATTTCTTCTCTCTGCCCAGGCTATCTAGGTAC[G/A]TTGAGTTT
GGGCTTTTGTTTAGTTGTTATTGTATGTGTATGAGTGTTTGTG[A/C]GGTTATATGTGTATAATCATCT
GCATGTTGTGCCCTTGGTGACCAGAAGAGGGTGTTCTATCCTCCAAAACTGGAGGCAGAGTCAGTTGCAA
ACCACCATGTGGGTGCTGAGAGTTGAACCTGGGTCTTCTGCAAGATCAGTAAATGCTCTTAACCGACCAG
AGCCTGTACACTGATGTGGGAGGAGATGGTGGGTGAAT[-/A]
AGTGT[G/T]CAGACTTGGAC[C/T]CTGCCCTT[T/C]CTTT
ACGCATTCCACTAACCATGGGATGGAAGGTTCTGCCTCTCAAAACCTTCAGTTCTGCATCTGTGAAATCA
TATTA[G/A]CAACCTTGAGTCTAAGTGGTCGGTGTGAGTATTAGACGTATATGCTC[G/A]GTTTATTA
ACTTCAGCTTCACGTCAG[A/G]GTAGGAGATGTATAAAGGGAAAAACAAAACAGAGCATAAGGTATAGT
AGCCTCGGGCAGCCATCTTCCAGTAACTCCCCAAAATGATGAACACAAAGGGAAAGAGGAGAGGCACCTG
GTATATGTTCTCTAGGCCTTTTAGGAAACATGGCATTGTTCCTTTGGCCACATACATGGGAATCTACAAG
AAGGGTGATATTGTGAACATCAAGGGAATGGGTACTGTTCAAAAAGGAATGCCCCATAAGTGCTACCACG
GCAAAACC[G/A]GAAGAGTCTACAATGTCACCCAGCATCCCGTAGGTATCATGGTAAACAAGCAAGTTA
AGGGCAAGATTCTGGCCAAGAGGACCAGTGTGTGGACTGAGCACATCAAGCACTCAAAGAGCAGAGACAG
CTTCCTGAAGCGGGTGAAGGAGAACGATCAGAAGAAAAAGGAAGCCAAAGAGAAGGGCACCTGGGTGCAG
CTGAAGCGCCAGCCTGCGCCACCCAGAGAAGCACACTTTGTGAGGACTAACGA[T/A]AAAGAGCCTGAG
CTGTTGGAGCCCATTCCATAC[G/A]AATTCATGGCCTAATGTACAAAAAT**[G/A]**AAATAAAGGACCAG
GACTGGAAAAAAAAAA
