# Supplementary material for: DNA binding specificities of the long zinc-finger recombination protein PRDM9
Source: Genome Biol. 2013 Apr 24;14(4):R35. doi: 10.1186/gb-2013-14-4-r35 (PMC4053984; doi:10.1186/gb-2013-14-4-r35)
Supplement: Additional file 4 — Figure S4. The PRDM9Cst binding site of Esrrg-1. The Additional material contains maps of all hotspots studied in this paper, their sequences, additional figures and tables highlighting specific points in the paper, and the sequences of the oligos used for mapping. [file gb-2013-14-4-r35-S4.PDF]

Additional file 4:

Figure S4. The PRDM9^{Cst} binding site of Esrrg-1.

(A) Binding of PCR-amplified fragments tiling Esrrg-1 to PRDM9^{Cst}.

Upper panel. Lanes 1–15: tiling-labeled fragments 1–15 incubated with PRDM9^{Cst}, as shown in Figure 3A. Additional bands present in all lanes are non-specific and probably caused by the presence of biotin-containing proteins in crude bacterial extract. Only the band in lane 8, indicated by an asterisk, was confirmed as specific by competition assay.

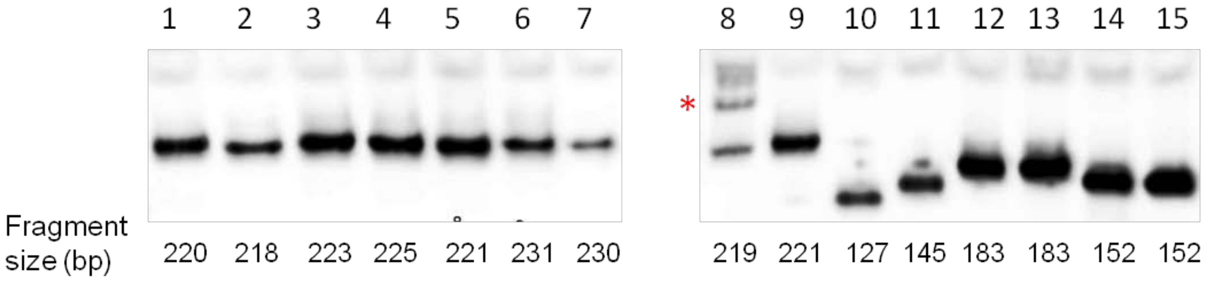

Lower panel. Competition assay for fragment 8. Lane 1, labeled fragment 8; lane 2, labeled fragment 8 + PRDM9^{Cst}; lane 3, labeled fragment 8 + PRDM9^{Cst} + excess of unlabeled fragment 8; lane 3, labeled fragment 8 + PRDM9^{Cst} + excess of unlabeled fragment 9; The second band in lane 1 is due to a misaligned fragment after denaturing; it disappears after adding bacterial extract. Unlabeled fragment 8 competes with the labeled fragment whereas fragment 9 does not.

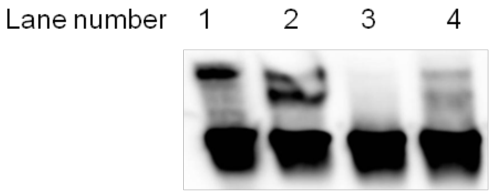

(B) Sequence of Esrrg1 with the flanking SNPs in bold and the PRDM9^{Cst} binding site in bold, underlined. SNPs between C57BL/6J and CAST/EiJ are shown.

GTTTGGGAATCAGGAGGCATTGTCGAGACCTTTGACGGG**[T/C]**GTGGCAGGACTTTCACTAGTCTAAAGGCACCTT
CCCTTGTAAGTGAGATGGTATGGGAGGGAAGGCAAGACGAAGCAGAGAGACCTTTCTGGGCAGAGACGTGAGGCTCA
CTTCCTTCTCCAGGGATCTCAGGATCCCAGGCTCTTCCCTGGGACCTCCTTTCTGCTTGCTCTTCCTTCCTAACTCT
GGGAATTATTTATCATCATCTTAGCATATCCAGACTGAAGGTGTGAGCTGAAGGGTAGAACATAGATGAATGCTAGT
TTATTTTCTGTGCCTGGACTACCTTAATGTATTGCTTCTACTCCTAACCAGGGCTTGGTTCTAATTTTATCCATGTT
CCTCAAGCCGGGTGAATGACGGCTACCCTGACCTCAGTATTGAGTTAGTATCAACTCATGAATAATTTGGAAGTATT
TGGAAGGAAACTGGATTGTTGGCTGATGCTTTGGCCTGTTTAAATATTTGTTCCTGCTTCCCTCACAAATGCACCCC
A[T/C]TCTTTATAATATCTAGAAGCAGGCTGAAGTGTCTCCCTCCCAGGAACTCCTTTATAGCTGTCAACCCAATT
GATACATGTTTTGGGTCCATGTCATGACTTTTAGTCAGCATGGCTTCTATTGGCTTACTCATCCTTCCTGGTAGCTC
CGTCCCACTCAATTAATGAAGCATTAGGTTGGCATGAAAAGTGCATGTGACACACTGGGGAATGGTGGTTCTTCTGT
TTCTAGGTTCTTGGGAAGCTGAGGCCAAAGAGTGACTTGAGTGCAGAAATCTGAAGCCAGCCTTGGTAGCATACTGA
GACCCTACCACAAGAAATCTGCCAAATAGAGAAAGAAAGAAACACAGAAAGAGAAGGTGGACTGAGTTAGATGGCTC
TGTCCTCTCCGTACATGGTCCTTTTAAGTATCACCATCAACTCTAGTTTGTGAAGTTTCCTGTTCTGGAAAATGTGA
AGTTGTGGTAGAGATGGTGGCAGTGGCTAGCACTTGCCCTGAGGAAAGCCTGGGTGTCCTCTGACGGTCCCATCTCC
CATTTGTAGCCTTCAGTCTTCCACACTAGCACTGCTAGGTTTTTGCTTTGTGAAATATATATTTCTCAATGATCCTG
AACTTTGTATAGAACTCTGCAGAGTCATCAGGTAGTGTCTAAAGGGGGTAACATAGGGGGGAAAGTTATAACCTCAA
CTTCACTAACAGTGACAGAGTAAATGTGTTCCTGTGTTATGT[G/A]TCTAAAGGAAGGAGGGTCTCCTCTTATCTC
CCAAATGTGTCTTTGACTTTGGTCTTTGCTTCAGCACTGAAATTCTTTCATAAGTAAAGAATTCTATTGCTACTTTA
ATGCCACTCAGAAACTGGCCTTTTCTAATGTTCCTAAGATTATGGCGCTCAGAATCCACCCCGCCCTC[T/G]CCAC
TCCCACTAGCTCGCAGTGACTGAGTTTAAACTAGCAGCCCCGGTTAAGGTATTGATTTTAACCTGAAGCCCAGAGGG
ACACAGGGCTGGAGTCAGCTGGACTCTTAAGCAGTCCTGGGAGACCAGA[G/A]TCATTTGCACATCGGAAAACAGC
TAATGGGCAGGGAATTAGGGCTTAGGTAGGCCTGTCCCTTCTGT[T/C]TTCCGTCCCTCACAGCTGCTATATGTCC
TGATAAAAAGGCAGGCAGACCCAAGACACCATATTTCATTTTCTTTCTCTCTCTCTCTCTTAAAAGTAAATTTTT
AAGAGTCTTTTATTTCTGATAAGGACACAGGTA[T/G]CTCAGATTGGCCTCAGACC[A/G]ACAAGAAAACTCATC
CCCCATCTCTGTCTCCCAAGGGCTGGGAATACAGACATGTTGCTTTGTTTATGTGACCCCCTAAGGGTCGAACCCAG
AGCCTCATTTTTG[G/A]TTAGCGAACACTACTCACTGAGCTGTACCCAAGGCTGAGTATTTTT[A/T]AAGGAAAC
TCAAGGAAGGGAAAATGATAGCTAGCCATTCTTTTTCACA[T/C]TGCAAAATTCTAGT[A/T]GTAGTGAA**<u>ATACT</u>**
<u>TTGCAAATATCAAGGCTCTAATACAAAT</u>CCTACTGGCGACATACTTGGAAGAGTATCCTCTTGTGTGTCATTCTGTA
TGGAATAGTCTTAAGTTTTTGGGCTGCCAATTACTTGTAAA[G/C]TTCTTCTTTAAAAATGT[T/C]CTATTAGCA
TGTATTCTCTATACATGAT[G/-]GGTGTGTGAAGTCTTCATGCATGGATGTGAGGTATTCTGATCACTGTTACCC
CGTCACCTTCTCTTCGCCCAGGCCCGCAGCTGCTCTTACCTCTCCCCTGCACAG[G/A][C/T]CTTGTAAGTCTCT
TCCTGTTTTGGACACCCAGCTGGGTTTCTGGCATATATCAGACGTCAGTATGAGCTGCTGTTCTGACCGGGGCAATC
CCATCCTTACCCAGTGTTTAGGACCCTAGTGGTTCCACCACACACCACCTTAAGCATCATCGTTTGTGTTTTGTTTC
ACTTTGTTTGGCTCTCTGCAGTGAATGAGGGCAGTGTTCACAGCAGGC
CTGGGTTGTCTTCACATTGCTTTCTCTCCATGACTCGCTAGGGTCAGAGGTCCCCGATAATGCGCTGTGTGACTCCG
GCCTGAGCCATTTGTGTGCCCTGGAGATCTGAGGGTAACAGGCAGGGTCAGCATAACCTGCCAGATTCTCAGAGACC
CCTCTGTGAGTTGTGGGAGGACAGTAAAAAAGCCTGTCACGGACAGAGTAACCTTCCGTCCATGGGACTGCTTTTCA
TCAGATGGTGCTCACTTTGCCCAAGTGTAACTGATGGGGTCTACAGTCAGTGCTGCACATGCTAATGTGTTCATTTG
ATTCTAGCAGGACACAAACCTCAGAGGGCCACTGCAGAAGAAGGGACATTCACCTCTATCTAAAACATTTCACTTCA
CTTTCTCCCTAAAAGTTTATAGGTGTTTTGAGCTTCTGACAACACAGGCTTAAAGATGTGTGTAGCTTGGCTCCTGT
CAGTGCTTTTCTGGACTCCTTAAGTTATTCCTCAAAGCTGTTCCTCTATTTAGGGAGGTAAAGTGTATTTCTTGTGT
ATCCTGAAAAAAAGAGGGATTTTATTTAATCCTTCCTTCTCTTTCTAGCCTATCTCC**[-/T]**TTTT
