# Supplementary material for: DNA binding specificities of the long zinc-finger recombination protein PRDM9
Source: Genome Biol. 2013 Apr 24;14(4):R35. doi: 10.1186/gb-2013-14-4-r35 (PMC4053984; doi:10.1186/gb-2013-14-4-r35)

Additional file 5:

Figure S5. Best alignment of the three PRDM9^{Cst} binding sites. The two 5′-adjacent nucleotides in the Hlx1 binding site and the three 5′-adjacent nucleotides in the Psmb9 binding site were added to the minimal site. The table shows the color key for positions with common nucleotides. The ZnF domain of PRDM9^{Cst} is shown below the graphic.

Color		Positions with common nucleotides
Red		Psmb9, Hlx1, Esrrg-1
Yellow		Psmb9, Hlx1
Cyan		Psmb9, Esrrg-1
Green		Hlx1, Esrrg-1

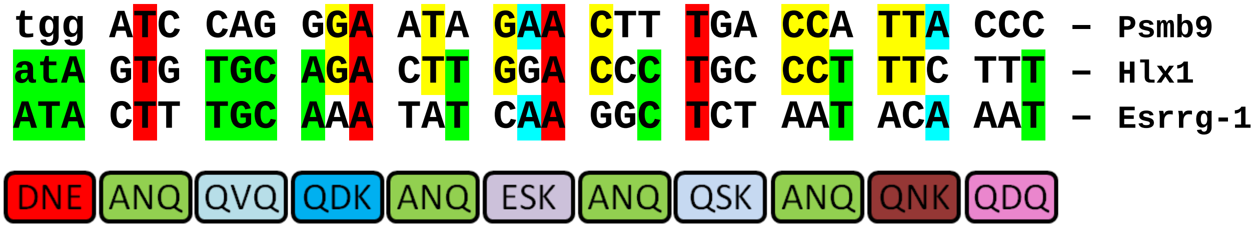

Supplement: Additional file 5 — Figure S5. Alignment of the three PRDM9Cst binding sites. The Additional material contains maps of all hotspots studied in this paper, their sequences, additional figures and tables highlighting specific points in the paper, and the sequences of the oligos used for mapping. [file gb-2013-14-4-r35-S5.PDF]
