# Supplementary material for: DNA binding specificities of the long zinc-finger recombination protein PRDM9
Source: Genome Biol. 2013 Apr 24;14(4):R35. doi: 10.1186/gb-2013-14-4-r35 (PMC4053984; doi:10.1186/gb-2013-14-4-r35)
Supplement: Additional file 6 — Table S1. Persikov algorithm predictions for 200 bp regions surrounding binding sites. The Additional material contains maps of all hotspots studied in this paper, their sequences, additional figures and tables highlighting specific points in the paper, and the sequences of the oligos used for mapping. [file gb-2013-14-4-r35-S6.PDF]

Additional file 6:

Table S1. Persikov algoritm predictions for 200 bp regions surrounding binding sites.

Hotspot	Specificity	SWM	Actual position	Predicted overlapping position	Strand	Rank	p value	Best predicted position for the opposite allele	Strand	p value
Hlx1	CAST	polynomial	92-122	92-123	lower	1	<0.0001	0-34	lower	<0.0001
		linear	92-122	none	-	-	-	63-97	lower	<0.0001
Esrrg-1	CAST	polynomial	96-128	93-124	upper	2	0.006	143-177	lower	0.006
		linear	96-128	91-122	lower	3	0.022	79-113	lower	0.002
Psmb9	CAST	polynomial	99-127	98-129	lower	5	0.024	55-89	lower	<0.0001
		linear	99-127	98-129	lower	1	0.003	63-97	upper	0.001
Pbx1	B6	polynomial	94-127	none	-	-	-	39-70	lower	0.009
		linear	94-127	91-125	lower	5	0.019	97-128	lower	0.008
