# Supplementary material for: DNA binding specificities of the long zinc-finger recombination protein PRDM9
Source: Genome Biol. 2013 Apr 24;14(4):R35. doi: 10.1186/gb-2013-14-4-r35 (PMC4053984; doi:10.1186/gb-2013-14-4-r35)
Supplement: Additional file 8 — Figure S6. Nucleotide substitution analysis of Hlx1. The Additional material contains maps of all hotspots studied in this paper, their sequences, additional figures and tables highlighting specific points in the paper, and the sequences of the oligos used for mapping. [file gb-2013-14-4-r35-S8.PDF]

Additional file 8:

Figure S6. Nucleotide substitution analysis of Hlx1.

(A) Kinetics of replacement of the Hlx1 binding site by a cold competitor. 0.5 pmol labeled oligo was incubated with bacterial extract containing PRDM9^{Cst} for 1 h, and then 20x excess of cold competitor was added and incubated for an increasing amount of time. The position of the specific band is indicated with an arrow. The fraction of the shifted band is indicated below.

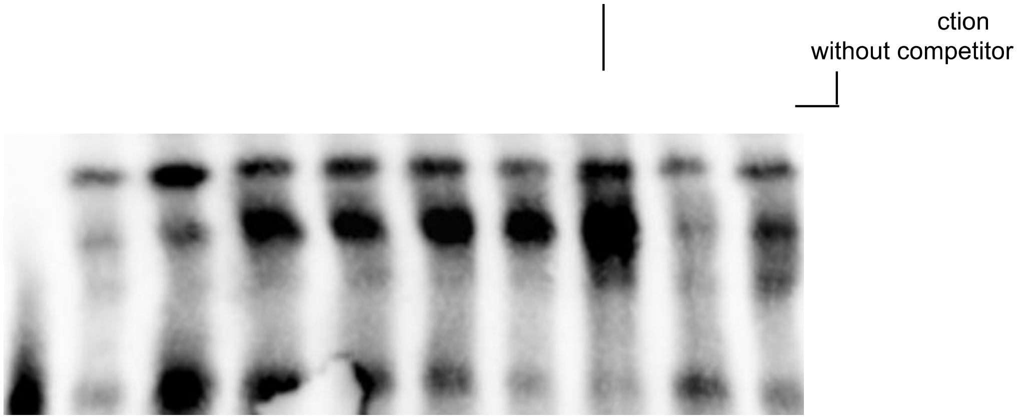

(B) Flow chart of the competition assay for testing the effect of single nucleotide replacements on DNA-PRDM9 binding.

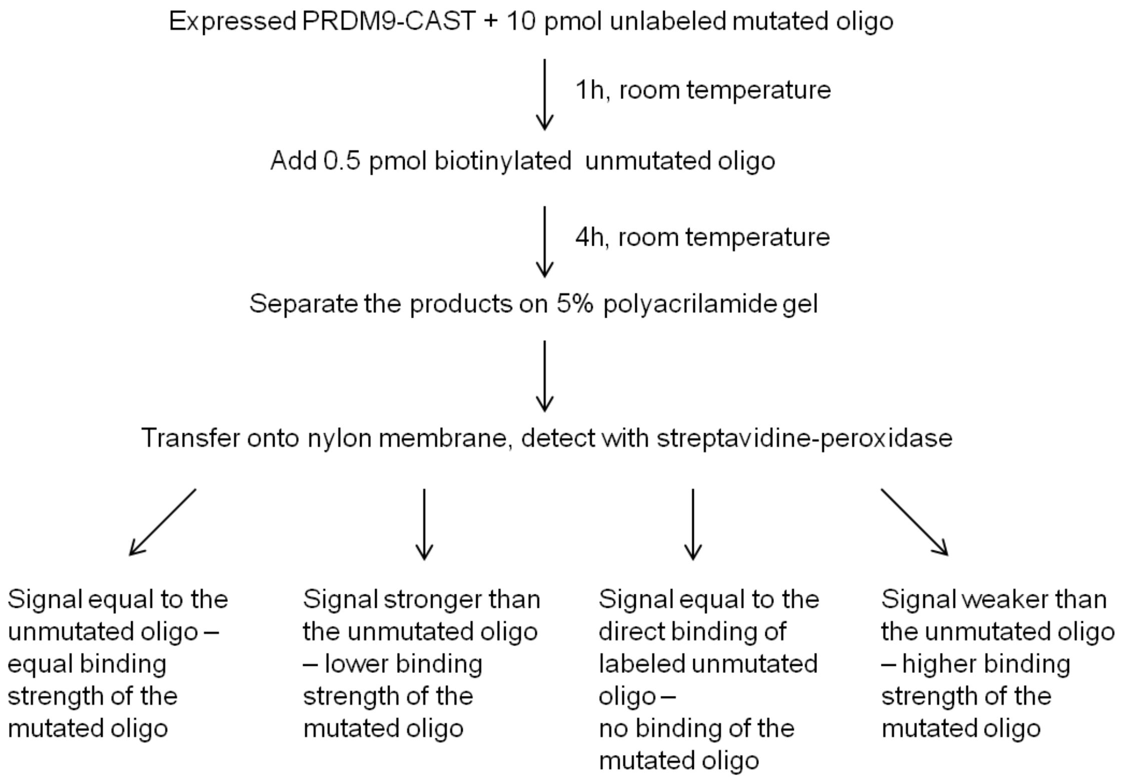

(C) Effect of single nucleotide substitutions on the binding strength between Hlx1 defined site ATAGTGTGCAGACTTGGACCCTGCCCTTTCTTTACG and PRDM9^{Cst}. Competition assay was performed as described in Materials and methods. The reaction component in each lane is indicated above each lane. Hlx1, unlabeled unmutated double-stranded oligo; Hlx1*, labeled unmutated double-stranded oligo; Scr – scrambled oligo with the same nucleotide composition as Hlx1; P9, expressed PRDM9^{Cst}. Position X: the consecutive nucleotide in the defined site mutated; the mutation is indicated immediately below the position for each of the three possible substitutions. The position of the shifted band is indicated by an arrow; the fraction of shifted band is shown below each picture.

a. Substitutions of nucleotides 1–3.

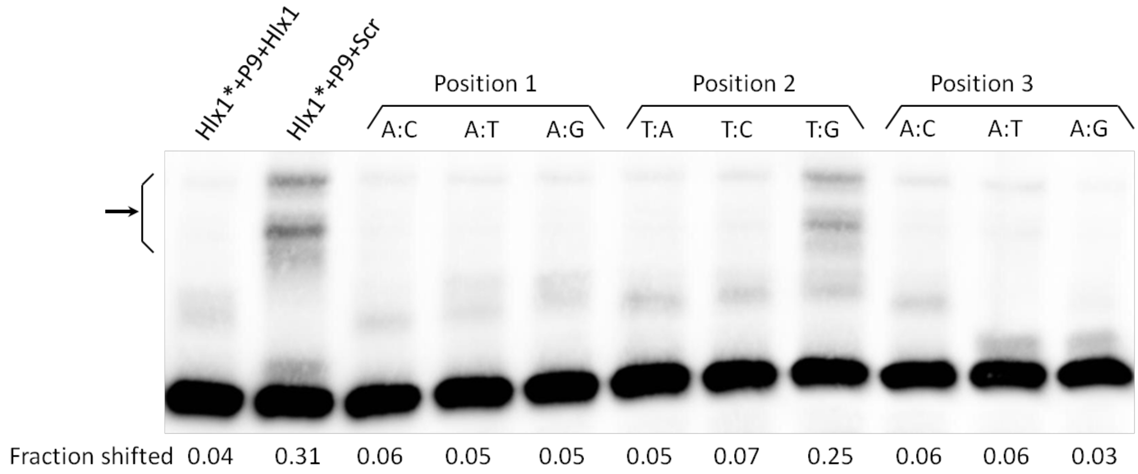

b. Substitutions of nucleotides 4–6.

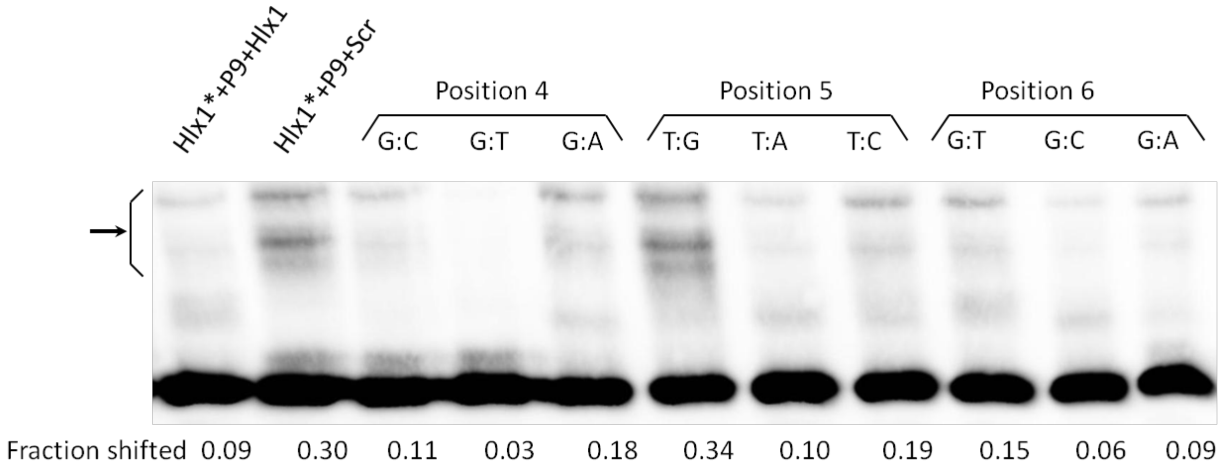

c. Substitutions of nucleotides 7–9.

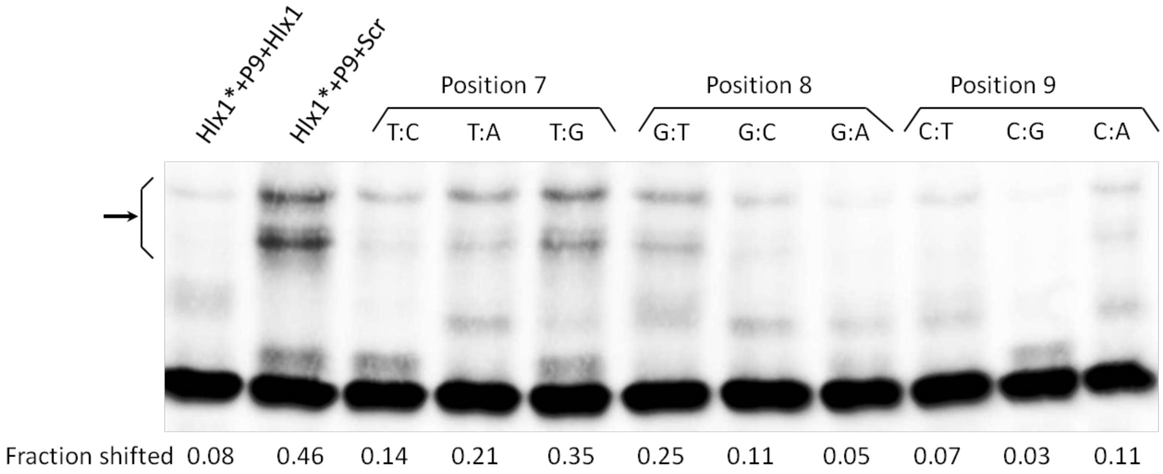

d. Substitutions of nucleotides 10–12.

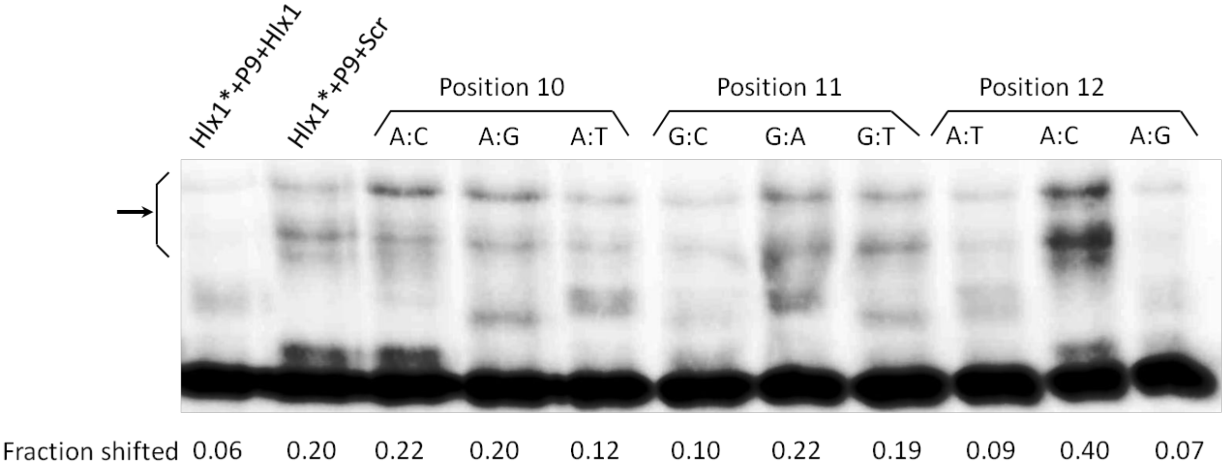

e. Substitutions of nucleotides 13–15.

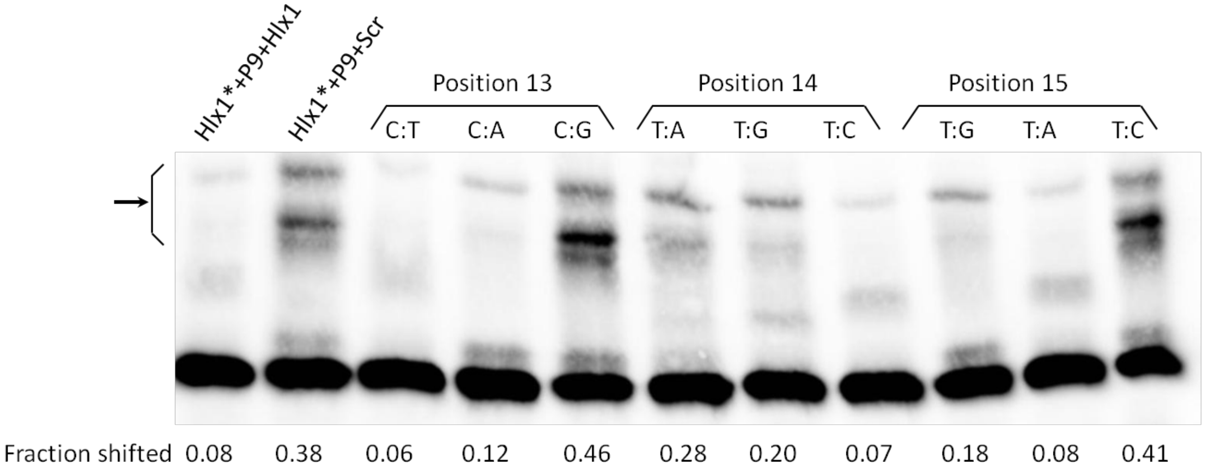

f. Substitutions of nucleotides 16–18.

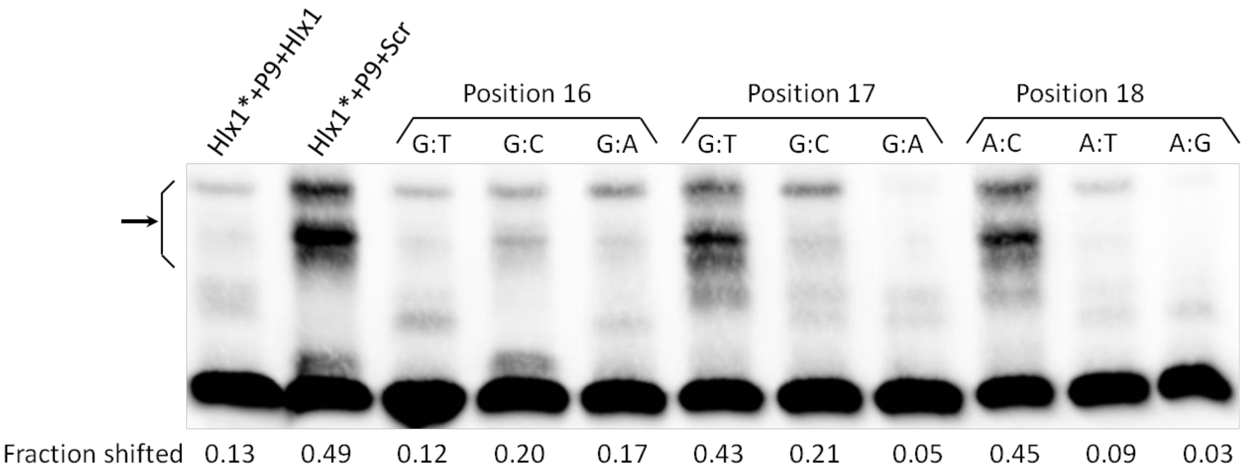

g. Substitutions of nucleotides 19–21.

h. Substitutions of nucleotides 22–24.

i. Substitutions of nucleotides 25–27.

j. Substitutions of nucleotides 28–30.

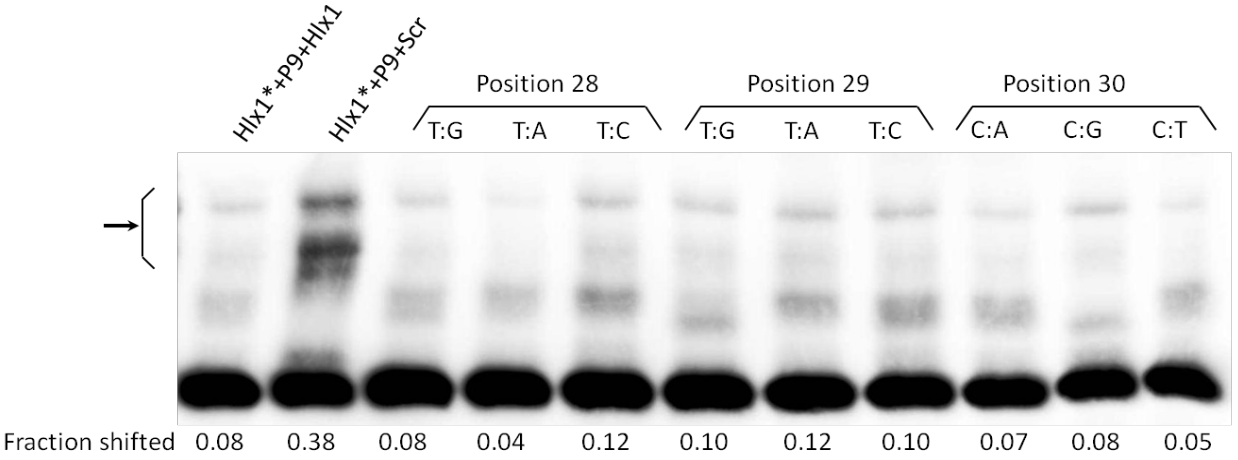

k. Substitutions of nucleotides 31–33.

I. Substitutions of nucleotides 34–36.
