# Supplementary material for: DNA binding specificities of the long zinc-finger recombination protein PRDM9
Source: Genome Biol. 2013 Apr 24;14(4):R35. doi: 10.1186/gb-2013-14-4-r35 (PMC4053984; doi:10.1186/gb-2013-14-4-r35)
Supplement: Additional file 9 — Table S3. Binding changes at each position along the Hlx1 binding site caused by a mutated nucleotide. The Additional material contains maps of all hotspots studied in this paper, their sequences, additional figures and tables highlighting specific points in the paper, and the sequences of the oligos used for mapping. [file gb-2013-14-4-r35-S9.PDF]

Additional file 9:

Table S3. Binding changes at each position along the Hlx1 binding site caused by a mutated nucleotide.

Position	1	2	3	4	5	6	7	8	9	10	11	12	13	14	15	16	17	18
	a	t	A	G	T	G	T	G	C	A	G	A	C	T	T	G	G	A
A	0.0	0.0	0.0	-0.4	0.0	0.0	-0.3	0.1	-0.1	0.0	-1.0	0.0	-0.1	-0.7	0.0	-0.1	0.2	0.0
C	-0.1	-0.1	-0.1	-0.1	-0.5	0.1	-0.2	-0.1	0.0	-1.0	-0.2	-1.0	0.0	0.0	-1.0	-0.2	-0.2	-0.9
T	0.0	0.0	-0.1	0.3	0.0	-0.3	0.0	-0.4	0.0	-0.4	-1.0	-0.2	0.1	0.0	0.0	0.0	-0.8	0.1
G	0.0	-0.8	0.0	0.0	-1.0	0.0	-0.7	0.0	0.1	-1.0	0.0	-0.1	-1.0	-0.4	-0.3	0.0	0.0	0.3
StDev	0.0	0.4	0.1	0.3	0.5	0.2	0.3	0.2	0.1	0.5	0.5	0.5	0.5	0.3	0.5	0.1	0.5	0.5

Position	19	20	21	22	23	24	25	26	27	28	29	30	31	32	33	34	35	36
	C	C	C	T	G	C	C	C	T	T	T	C	T	T	T	a	a	t
A	-0.1	-0	0.07	0.01	0.1	-0.3	-0.1	-0.1	0.09	0.13	-0.1	0.05	0.08	0.15	0.13	0	0	0.2
C	0	0	0	-0.1	-0.1	0	0	0	0.19	-0.1	-0.1	0	0.12	0.13	0.14	0.18	0.11	0.18
T	-0.3	0.01	0.06	0	-0.3	0.02	-0.2	0.15	0	0	0	0.11	0	0	0	0.12	0.12	0
G	-0.5	-0.1	0.06	-0.5	0	0.03	-0.4	0.04	0.1	0.01	-0	-0	0.12	0.09	0.09	0.12	-0.9	0.16
StDev	0.23	0.05	0.03	0.23	0.17	0.17	0.16	0.12	0.08	0.1	0.05	0.06	0.06	0.07	0.06	0.08	0.5	0.09
