# Supplementary material for: DNA binding specificities of the long zinc-finger recombination protein PRDM9
Source: Genome Biol. 2013 Apr 24;14(4):R35. doi: 10.1186/gb-2013-14-4-r35 (PMC4053984; doi:10.1186/gb-2013-14-4-r35)

Additional file 10:

Figure S7. Effect of Mg^{2+} on PRDM9-DNA binding. EMSA was performed as described in Materials and methods, except that $MgCl_2$ was added to the reaction mixtures to final concentrations shown in the figure. The chart below the pictures shows a quantitation of the effect of Mg2+ with standard deviations. The fraction of bound oligo was normalized using the formula

$$NB = (FB_{[MgCl2]} - FB_{compete})/(FB_{oligo} - FB_{compete}),$$

where NB is Normalized Binding, and $FB_{[MgCl2]}$, $FB_{compete}$, and FB_{oligo} are the fractions of bound oligo in the test samples (lanes 3–8), competitor (lane 2) and no $MgCl_2$ (lane 1), respectively.

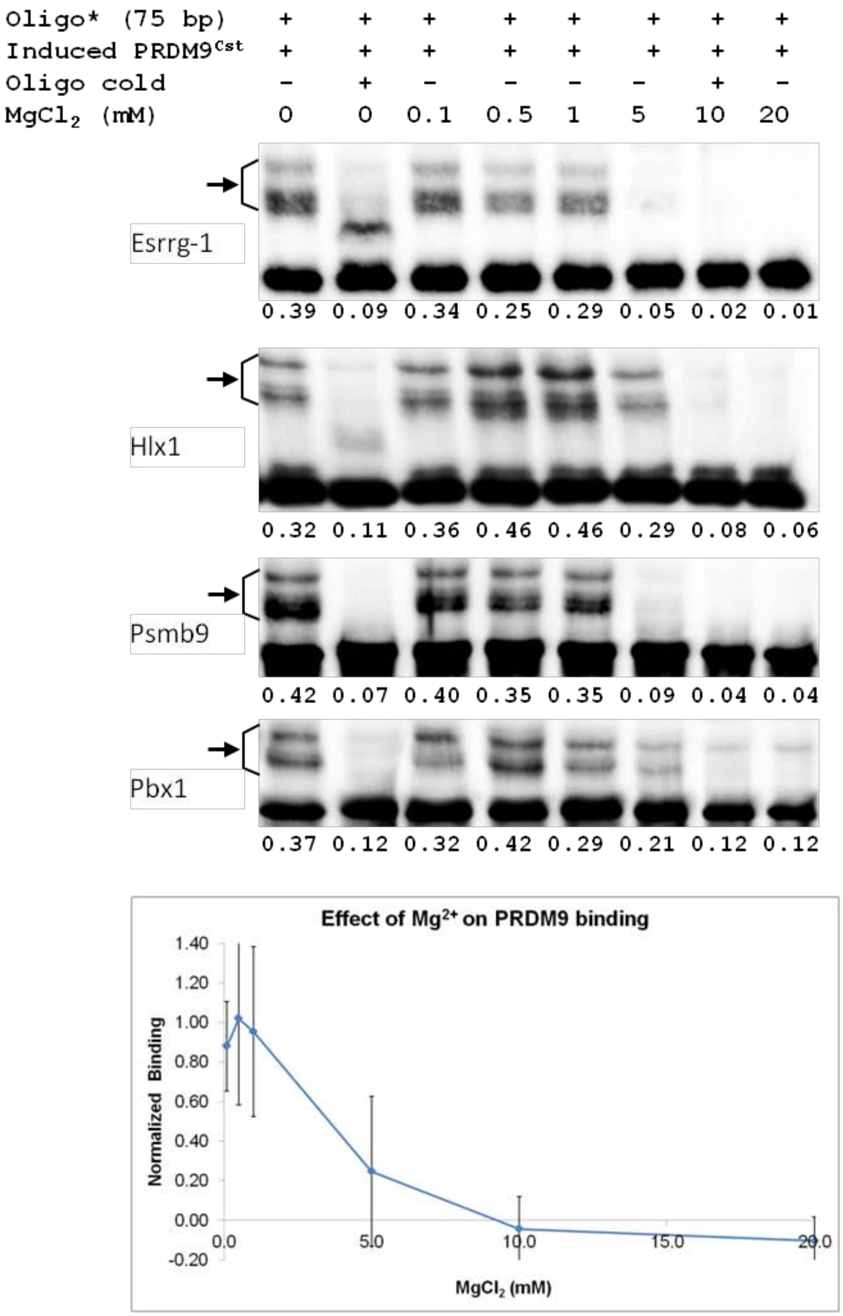

Supplement: Additional file 10 — Figure S7. Effect of Mg2+ on PRDM9-DNA binding. The Additional material contains maps of all hotspots studied in this paper, their sequences, additional figures and tables highlighting specific points in the paper, and the sequences of the oligos used for mapping. [file gb-2013-14-4-r35-S10.PDF]
