# Supplementary material for: DNA binding specificities of the long zinc-finger recombination protein PRDM9
Source: Genome Biol. 2013 Apr 24;14(4):R35. doi: 10.1186/gb-2013-14-4-r35 (PMC4053984; doi:10.1186/gb-2013-14-4-r35)
Supplement: Additional file 11 — Additional experimental procedures: oligos used for analysis of binding. The Additional material contains maps of all hotspots studied in this paper, their sequences, additional figures and tables highlighting specific points in the paper, and the sequences of the oligos used for mapping. [file gb-2013-14-4-r35-S11.PDF]

Additional file 11:

ADDITIONAL EXPERIMENTAL PROCEDURES

Oligos used for *Prdm9* cloning

```
Prdm9F-HisB – CGAGCTCGAGAATGAACACCAACAAGCTGGAAG
Prdm9R-HisB – AGCCAAGCTTTTACTTCTCTCTTGTATGTGTCCT
```

Oligos used for *Prdm9* sequencing

```
pBAD/HisBf - TCGCAACTCTCTACTGTTTCTCC
Prdm9F1    - GAAGTGCCAGAATTTCTTCATC
Prdm9F2    - GATGAAGAAAGGATTCACAGC
pBAD/HisBr - GTATCAGGCTGAAAATC
```

Oligos used for analysis of binding

Oligos for amplification of PCR products tiling Hlx1

```
Hlx1left1f     TTCAGCGAGCACAGACTCTT
Hlx1left1r     CCAAGCATGGTTCATCAAAA
Hlx1left2f     AAATTAATGTTCGATGCTTGTGG
Hlx1left2r     GACAAGCTGGGAGAGGGAGT
Hlx1left3f     AAGTTCCTCTCCATCATCTCCCT
Hlx1left3r     ATTCTGCCTGGAAAGGAGTG
Hlx1left4f     GTTAAATGCCCACCCTGAAC
Hlx1left4r     AAGGGTAATGATTTGGGGAAA
Hlx1middle1f   AGCCCTTCCTTATCACAGCA
Hlx1middle1r   AAGCCCAAACTCAACGTACC
Hlx1middle2f   CGTTGAGTTTGGGCTTTTGT
Hlx1middle2r   CCATCCCATGGTTAGTGGAA
Hlx1middle3f   CCACTAACCATGGGATGGAA
Hlx1middle3r   GGGGAGTTACTGGAAGATGG
Hlx1right1f    GCTGGTACCCGTTCGTTTAT
Hlx1right1r    GTCTCTTCCAGGAAAGTTTCAA
```

Oligos for final mapping of Hlx1 binding site

```
30bpf          GTGTGCAGACTTGGACCCTGCCCTTTCTTT
30bpr          AAAGAAAGGGCAGGGTCCAAGTCTGCACAC
31bpl          AGTGTGCAGACTTGGACCCTGCCCTTTCTTT
31bpr          AAAGAAAGGGCAGGGTCCAAGTCTGCACACT
32bpf          TAGTGTGCAGACTTGGACCCTGCCCTTTCTTT
32bpr          AAAGAAAGGGCAGGGTCCAAGTCTGCACACTA
33bpf          ATAGTGTGCAGACTTGGACCCTGCCCTTTCTTT
```

33bpr	AAAGAAAGGGCAGGGTCCAAGTCTGCACACTAT
36bpf	ATAGTGTGCAGACTTGGACCCTGCCCTTTCTTTACG
36bpr	CGTAAAGAAAGGGCAGGGTCCAAGTCTGCACACTAT
39bpf	TGAATAGTGTGCAGACTTGGACCCTGCCCTTTCTTTACG
39bpr	CGTAAAGAAAGGGCAGGGTCCAAGTCTGCACACTATTCA
36bpCastf	ATAAGTGTTCAGACTTGGACTCTGCCCTTCCTTTAC
36bpCastr	GTAAAGGAAGGGCAGAGTCCAAGTCTGAACACTTAT
75bpf	GTGGGAGGAGATGGTGGGTGAATAGTGTGCAGACTTGGACCCTGCCCTTTC TTTACGCATTCCACTAACCATGGG

Oligos for amplification of PCR products tiling Pbx1

Pbx1-1f	CTGAGCAGATACTATCATATGGAGAAC
Pbx1-1r	GCCTTCATATGTCAGTTTCTATCTTTT
Pbx1-2f	TGTCCAGTTTAAGGGAGCAAA
Pbx1-2r	CATACTCCAGTCATCCTCAAAGG
Pbx1-3f	TTGGTAAGTGTTTTATCTTGATGTGA
Pbx1-3r	AAAGCACTACCTTTCTTACCAGTCTC
Pbx1-4f	TGGTTAAGAGCAAAGCTAGCAA
Pbx1-4r	TGCTGAAGTCACAACAACAACA
Pbx1-5f	AAGAAAATCACGGCCTTCTG
Pbx1-5r	AGGTGCTCTGTGACCAATGA

Oligos for final mapping of Pbx1 binding site

Pbx1f1sp	CTGAGCAGATACTATCATATGGAGAACTTACAAAGGTAGGACGTAAATGG
Pbx1r1sp	CCATTTACGTCCTACCTTTGTAAGTTCTCCATATGATAGTATCTGCTCAG
Pbx1f2sp	ACTTACAAAGGTAGGACGTAAATGGAGTGTTTACACCACCGTACTAGAGG
Pbx1r2sp	CCTCTAGTACGGTGGTGTAAACACTCCATTTACGTCCTACCTTTGTAAGT
Pbx1f3sp	AGTGTTTACACCACCGTACTAGAGGAAGTCAAAGTGTGTTCTCATTTTGA
Pbx1r3sp	TCAAAATGAGAACACACTTTGACTTCCTCTAGTACGGTGGTGTAAACACT
Pbx1f4sp	AAGTCAAAGTGTGTTCTCATTTTGAAACAGGTTAGACATTTCTTGTAAAC
Pbx1r4sp	GTTTACAAGAAATGTCTAACCTGTTTCAAAATGAGAACACACTTTGACTT
Pbx1f5sp	AACAGGTTAGACATTTCTTGTAAACACAAACTTACCATGTAATCTATAAA
Pbx1r5sp	TTTATAGATTACATGGTAAGTTTGTGTTTACAAGAAATGTCTAACCTGTT
29bpf	GAGAACTTACAAAGGTAGGACGTAAATGG
29bpr	CCATTTACGTCCTACCTTTGTAAGTTCTC
31bpf	GAGAACTTACAAAGGTAGGACGTAAATGGAG
31bpr	CTCCATTTACGTCCTACCTTTGTAAGTTCTC
33bpf	GAGAACTTACAAAGGTAGGACGTAAATGGAGTG
33bpr	CACTCCATTTACGTCCTACCTTTGTAAGTTCTC
34bpf	GAGAACTTACAAAGGTAGGACGTAAATGGAGTGT
34bpr	ACACTCCATTTACGTCCTACCTTTGTAAGTTCTC
37bpf	GAGAACTTACAAAGGTAGGACGTAAATGGAGTGTTTA
37bpr	TAAACACTCCATTTACGTCCTACCTTTGTAAGTTCTC
40bpf	ATGGAGAACTTACAAAGGTAGGACGTAAATGGAGTGTTTA
40bpr	TAAACACTCCATTTACGTCCTACCTTTGTAAGTTCTCCAT

Oligos for amplification of PCR products tiling Esrrg-1

Esrrg1f	AAGCCAGCCTTGGTAGCATA
Esrrg1r	TTTCCTCAGGGCAAGTGCTA
Esrrg2f	AATGTGAAGTTGTGGTAGAGATGG
Esrrg2r	CCCCCTATGTTACCCCCTTT
Esrrg3f	TCTGCAGAGTCATCAGGTAGTG
Esrrg3r	GCCAGTTTCTGAGTGGCATT
Esrrg4f	CCACTCAGAAACTGGCCTTT
Esrrg4r	CTGCCCATTAGCTGTTTTCC
Esrrg5f	GGCCTCAGACCAACAAGAAA
Esrrg5r	TTTGCAATGTGAAAAAGAATGG
Esrrg6f	TAAGTTTTTGGGCTGCCAAT
Esrrg6r	CAGCTGGGTGTCCAAAACAG
Esrrg7f	TAAGGTGGTGTGTGGTGGAA
Esrrg7r	CCCTAGTGGTTCCACCACAC
Esrrg8f	TGCACATCGGAAAACAGCTA
Esrrg8r	GAGACAGAGATGGGGGATGA
Esrrg9f	CCCTAGCGAGTCATGGAGAG
Esrrg9r	TGCTTTCTCTCCATGACTCG
Esrrg10f	CTGTCCGTGACAGGCTTTTT
Esrrg10r	TGTGAGTTGTGGGAGGACAG
Esrrg11f	TTCTAGCAGGACACAAACCTCA
Esrrg11r	CAGGAGCCAAGCTACACACA
Esrrg12f	TGTGTGTAGCTTGGCTCCTG
Esrrg12r	TAGGCTAGAAAGAGAAGGAAGGA
Esrrg13f	TGTGAGTTGTGGGAGGACAG
Esrrg13r	AGTGGCCCTCTGAGGTTTGT
Esrrg14f	TTCTAGCAGGACACAAACCTCA
Esrrg14r	CAGGAGCCAAGCTACACACA
Esrrg15f	TGTGTGTAGCTTGGCTCCTG
Esrrg15r	TAGGCTAGAAAGAGAAGGAAGGA

Oligos for final mapping of Esrrg-1 binding site

Esrrg80f	AAAATTCTAGTAGTAGTGAAATACTTTGCAAATATCAAGGCTCTAATACAA ATCCTACTGGCGACATACTTGGAAGAGTA
Esrrg80r	TACTCTTCCAAGTATGTCGCCAGTAGGATTTGTATTAGAGCCTTGATATTT GCAAAGTATTTCACTACTACTAGAATTTT
30bpf	ATACTTTGCAAATATCAAGGCTCTAATACA
30bpr	TGTATTAGAGCCTTGATATTTGCAAAGTAT
33bpf	ATACTTTGCAAATATCAAGGCTCTAATACAAAT
33bpr	ATTTGTATTAGAGCCTTGATATTTGCAAAGTAT
36bpf	GAAATACTTTGCAAATATCAAGGCTCTAATACAAAT
36bpr	ATTTGTATTAGAGCCTTGATATTTGCAAAGTATTTC

Oligos for initial mapping of Psmb9 binding site

Psmb9-1f	CTAGCGCCACGTGGTATATG
Psmb9-1r	ACAACAGAACTGTCAAGAAATAACA
Psmb9-2f	TTCTTGACAGTTCTGTTGTACATTTG
Psmb9-2r	GGGAAACTGAGGGTGAGAAA
Psmb9-3f	TTTCTCACCCTCAGTTTCCC
Psmb9-3r	CAACAAGCTGAAATGCAAACGCATTCAC

Oligos for final mapping of Psmb9 binding site

30rf	CCAGGGAATAGAACTTTGACCATTACCCAC
30rr	GTGGGTAATGGTCAAAGTTCTATTCCCTGG
28rf	CCAGGGAATAGAACTTTGACCATTACCC
28rr	GGGTAATGGTCAAAGTTCTATTCCCTGG
25rf	CCAGGGAATAGAACTTTGACCATTA
25rr	TAATGGTCAAAGTTCTATTCCCTGG
32lf	GGATCCAGGGAATAGAACTTTGACCATTACCC
32lr	GGGTAATGGTCAAAGTTCTATTCCCTGGATCC
30lf	ATCCAGGGAATAGAACTTTGACCATTACCC
30lr	GGGTAATGGTCAAAGTTCTATTCCCTGGAT
