# Supplementary material for: DNA binding specificities of the long zinc-finger recombination protein PRDM9
Source: Genome Biol. 2013 Apr 24;14(4):R35. doi: 10.1186/gb-2013-14-4-r35 (PMC4053984; doi:10.1186/gb-2013-14-4-r35)
Supplement: Additional file 12 — Sequences of Prdm9Dom2 and Prdm9Cst cDNA cloned in pBAD/HisB. The Additional material contains maps of all hotspots studied in this paper, their sequences, additional figures and tables highlighting specific points in the paper, and the sequences of the oligos used for mapping. [file gb-2013-14-4-r35-S12.PDF]

**Additional file 12:**

**Sequences of *Prdm9*$^{Dom2}$ and *Prdm9*$^{Cst}$ cDNA cloned in pBAD/HisB.** The leader sequence of pBAD/HisB including are uncolored; the initiation ATG and the 6-His tag are underlined. Prdm9 full length cDNA sequence is outlined in yellow.

Correct clone of Prdm9$^{Dom2}$ in pBAD/HisB:

ATGGGGGGTTCTCATCATCATCATCATCATGGTATGGCTAGCATGACTGGTGGACAGCAAATGGGTCGGGATCTGTACGACGATGA
CGATAAGGATCCGAGCTCGAGAATGAACACCAACAAGCTGGAAGAAAATAGTCCTGAAGAAGATACAGGGAAATTCGAGTGGAAAC
CCAAGGTCAAAGATGAATTCAAAGACATTTCCATATACTTCTCCAAAGAAGAATGGGCAGAGATGGGAGAGTGGGAGAAAATTCGC
TATAGAAATGTGAAAAGGAACTATAAAATGCTGATTTCAATAGGTCTCAGAGCCCCTAGACCAGCTTTCATGTGTTACCAAAGGCA
AGCAATGAAACCCCAAATAAATGACAGTGAGGATTCTGATGAAGAGTGGACACCTAAGCAACAAGTCAGTCCTCCTTGGGTGCCTT
TCCGGGTGAAGCACAGTAAACAGCAGAAGGAATCATCTAGAATGCCATTTAGTGGTGAATCTAATGTGAAGGAAGGGTCTGGAATA
GAAAATTTGCTGAATACAAGTGGCTCAGAACACGTCCAGAAACCAGTGTCCTCCCTTGAAGAAGGAAATACCTCTGGACAGCACTC
TGGGAAAAAACTGAAACTTAGGAAAAAGAACGTTGAAGTGAAAATGTACAGGCTGCGAGAGAGAAAGGGCCTTGCCTATGAGGAGG
TCAGCGAGCCTCAGGATGATGACTATCTCTATTGTGAGAAGTGCCAGAATTTCTTCATCGACAGTTGTCCCAACCATGGGCCTCCT
TTATTTGTAAAAGACAGTATGGTGGACAGGGGGCATCCCAACCACTCAGTCCTCAGTCTGCCCCCTGGGCTAAGAATTAGTCCATC
GGGCATCCCTGAAGCTGGACTTGGAGTATGGAATGAAGCATCTGATCTACCAGTCGGTCTGCACTTTGGCCCCTATGAGGGTCAGA
TCACAGAGGATGAAGAGGCAGCCAACAGTGGTTACTCCTGGCTGATTACCAAGGGAAGAAACTGCTATGAGTATGTGGATGGACAG
GACGAGTCCCAAGCCAACTGGATGAGGTATGTGAACTGTGCCCGGGATGATGAAGAGCAGAACCTGGTAGCCTTTCAATATCACAG
GAAAATCTTCTATCGAACCTGCCGGGTTATCAGACCAGGTTGTGAGCTTCTGGTCTGGTATGGGGATGAGTACGGCCAGGAACTGG
GCATTAAGTGGGGAAGCAAGATGAAGAAAGGATTCACAGCAGGAAGAGAACTAAGGACAGAAATTCATCCTTGTCTTTTGTGCTCT
TTGGCCTTCTCAAGTCAGAAATTCCTCACTCAACATATGGAATGGAATCATCGCACTGAAATCTTCCCAGGAACATCTGCAAGAAT
AAATCCTAAACCAGGAGATCCCTGTTCAGATCAGCTTCAGGAACAACATGTTGATTCACAGAACAAAAATGACAAGGCCAGCAATG
AAGTAAAAAGAAAATCCAAACCCAGGCAGAGGATTTCAACAACCTTTCCCAGCACACTCAAAGAACAAATGAGATCTGAGGAAAGT
AAGAGAACTGTGGAAGAGCTCAGAACAGGCCAGACAACAAATACAGAGGACACAGTCAAATCATTTATTGCATCAGAAATCTCAAG
TATTGAAAGACAATGTGGGCAATATTTCAGTGATAAGTCAAATGTCAATGAGCACCAGAAGACACACACAGGGGAGAAGCCCTATG
TTTGCAGGGAGTGTGGGCGGGGCTTTACACAGAACTCACACCTCATCCAGCACCAGAGGACACACACAGGGGAGAAGCCCTATGTT
TGCAGGGAGTGTGGGCGGGGCTTTACACAGAAGTCAGACCTCATCAAGCACCAGAGGACACACACAGGGGAGAAGCCCTATGTTTG
CAGGGAGTGTGGGCGGGGCTTTACACAGAAGTCAGACCTCATCAAGCACCAGAGGACACACACAGGGGAGAAGCCCTATGTTTGCA
GGGAGTGTGGGCGGGGCTTTACACAGAAGTCAGTCCTCATCAAGCACCAGAGGACACACACAGGGGAGAAGCCCTATGTTTGCAGG
GAGTGTGGGCGGGGCTTTACACAGAAGTCAGTCCTCATCAAGCACCAGAGGACACACACAGGGGAGAAGCCCTATGTTTGCAGGGA
GTGTGGGCGGGGCTTTACAGCGAAGTCAGTCCTCATCCAGCACCAGAGGACACACACAGGGGAGAAGCCCTATGTTTGCAGGGAGT
GTGGGCGGGGCTTTACAGCGAAGTCAAACCTCATCCAGCACCAGAGGACACACACAGGGGAGAAGCCCTATGTTTGCAGGGAGTGT
GGGCGGGGCTTTACAGCGAAGTCAGTCCTCATCCAGCACCAGAGGACACACACAGGGGAGAAGCCCTATGTTTGCAGGGAGTGTGG
GCGGGGCTTTACAGCGAAGTCAGTCCTCATCCAGCACCAGAGGACACACACAGGGGAGAAGCCCTATGTTTGCAGGGAGTGTGGGC
GGGGCTTTACACAGAAGTCAAACCTCATCAAGCACCAGAGGACACACACAGGGGAGAAGCCCTATGTTTGCAGGGAGTGTGGGTGG
GGCTTTACACAGAAGTCAGACCTCATCCAGCACCAGAGGACACATACAAGAGAGAAGTAA

Correct clone of Prdm9$^{Cst}$ in pBAD/HisB:

ATGGGGGGTTCTCATCATCATCATCATCATGGTATGGCTAGCATGACTGGTGGACAGCAAATGGGTCGGGATCTGTACGACGATGA
CGATAAGGATCCGAGCTCGAGAATGAACACCAACAAGCTGGAAGAAAATAGTCCTGAAGAAGATACAGGGAAATTCGAGTGGAAAC
CCAAGGTCAAAGATGAATTCAAAGACATTTCCATATACTTCTCCAAAGAAGAATGGGCAGAGATGGGAGAGTGGGAGAAAATTCGC

TATAGAAATGTGAAAAGGAACTATAAAATGCTGATTTCAATAGGTCTCAGAGCCCCTAGACCAGCTTTCATGTGTTACCAAAGGCA
AGCAATGAAACCCCAAATAAATGACAGTGAGGATTCTGATGAAGAGTGGACACCTAAGCAACAAGTCAGTCCTCCTTGGGTGCCTT
TCCGGGTGAAGCACAGTAAACAGCAGAAGGAATCATCTAGAATGCCATTTAGTGGTGAATCTAATGTGAAGGAAGGGTCTGGAATA
GAAAATTTGCTGAATACAAGTGGCTCAGAACACGTCCAGAAACCAGTGTCCTCCCTTGAAGAAGGAAATACCTCTGGACAGCACTC
TGGGAAAAAACTGAAACTTAGGAAAAAGAACGTTGAAGTGAAAATGTACAGGCTGCGAGAGAGAAAGGGCCTTGCCTATAAGGAGG
TCAGCGAGCCTCAGGATGATGACTATCTCTATTGTGAGAAGTGCCAGAATTTCTTCATCGACAGTTGTCCCAACCATGGGCCTCCT
TTATTTGTAAAAGACAGTATGGTGGACAGGGGGCATCCCAACCACTCAGTCCTCAGTCTGCCCCCTGGGCTAAGAATTAGTCCATC
GGGCATCCCTGAAGCTGGACTTGGAGTATGGAATGAAGCATCTGATCTACCAGTCGGTCTGCACTTTGGCCCCTATGAGGGTCAGA
TCACAGAGGATGAAGAGGCAGCCAACAGTGGTTACTCCTGGCTGATTACCAAGGGAAGAAACTGCTATGAGTATGTGGATGGACAG
GACGAGTCCCAAGCCAACTGGATGAGGTATGTGAACTGTGCCCGGGATGATGAAGAGCAGAACCTGGTAGCCTTTCAATATCACAG
GAAAATCTTCTATCGAACCTGCCGGGTTATCAGACCAGGTTGTGAGCTTCTGGTCTGGTATGGGGATGAGTACGGCCAGGAACTGG
GCATTAAGTGGGGAAGCAAGATGAAGAAAGGATTCACAGCAGGAAGAGAACTAAGGACAGAAATTCATCCTTGTCTTTTGTGCTCT
TTGGCCTTCTCAAGTCAGAAATTCCTCACTCAACATATGGAATGGAATCATCGCACTGAAATCTTCCCAGGAACATCTGCAAGAAT
AAATCCTAAACCAGGAGATCCCTGTTCAGATCAGCTTCAGGAACAACATGTTGATTCACAGAACAAAAATGACAAGGCCAGCAATG
AAGTAAAAAGAAAATCCAAACCCAGGCAGAGGATTTCAACAACCTTTCCCAGCACACTCAAAGAACAAATGAGATCTGAGGAAAGT
AAGAGAACTGTGGAAGAGCTCAGAACAGGCCAGACAACAAATACAGAGGACACAGTCAAATCATTTATTGCATCAGAAATCTCAAG
TATTGAAAGACAATGTGGGCAATATTTCAGTGATAAGTCAAATGTCAATGAGCACCAGAAGACACACACAGGGGAGAAGCCCTATG
TTTGCAGGGAGTGTGGGCGGGGCTTTACAGCGAAGTCAAACCTCATCCAGCACCAGAGGACACACACAGGGGAGAAGCCCTATGTT
TGCAGGGAGTGTGGGCGGGGCTTTACACAGAAGTCAGTCCTCATCCAGCACCAGAGGACACACACAGGGGAGAAGCCCTATGTTTG
CAGGGAGTGTGGGCGGGGCTTTACACAGAAGTCAGACCTCATCAAGCACCAGAGGACACACACAGGGGAGAAGCCCTATGTTTGCA
GGGAGTGTGGGCGGGGCTTTACAGCGAAGTCAAACCTCATCCAGCACCAGAGGACACACACAGGGGAGAAGCCCTATGTTTGCAGG
GAGTGTGGGCGGGGCTTTACAGAGAAGTCAAGCCTCATCAAGCACCAGAGGACACACACAGGGGAGAAGCCCTATGTTTGCAGGGA
GTGTGGGTGGGGCTTTACAGCGAAGTCAAACCTCATCCAGCACCAGAGGACACACACAGGGGAGAAGCCCTATGTTTGCAGGGAGT
GTGGGCGGGGCTTTACACAGAAGTCAAGCCTCATCAAGCACCAGAGGACACACACAGGGGAGAAGCCCTATGTTTGCAGGGAGTGT
GGGCGGGGCTTTACAGCGAAGTCAAACCTCATCCAGCACCAGAGGACACACACAGGGGAGAAGCCCTATGTTTGCAGGGAGTGTGG
GTGGGGCTTTACACAGAAGTCAAACCTCATCAAGCACCAGAGGACACACACAGGGGAGAAGCCCTATGTTTGCAGGGAGTGTGGGT
GGGGCTTTACACAGAAGTCAGACCTCATCCAGCACCAGAGGACACATACAAGAGAGAAGTAA
