# Supplementary material for: DNA binding specificities of the long zinc-finger recombination protein PRDM9
Source: Genome Biol. 2013 Apr 24;14(4):R35. doi: 10.1186/gb-2013-14-4-r35 (PMC4053984; doi:10.1186/gb-2013-14-4-r35)

**Additional file 13:**

**Figure S8. PRDM9 binds to its targets in an allele-specific manner.** Western blotting with anti-PRDM9 antibodies of protein-DNA complexes isolated on streptavidin beads. PRDM9 alleles were expressed in *E. coli* and bound to biotynilated oligos representing binding sites as described for the EMSA experiments. The complexes were then isolated on streptavidin beads and washed three times, and the bound proteins were released from the beads by adding 0.1% SDS. The specificity of PRDM9 binding was detected on western blots using antibodies against the C-terminal part of PRDM9.

The position of the full length protein is indicated by black arrows. Expression of both alleles in *E.coli* is associated with substantial protein degradation, as seen by the multiple bands detected by the C-terminal antibody. Note that all fragments longer than 30 kDa detected by the antibody (lanes 13 and 18) are represented in the protein-DNA complexes (lanes 3, 7, 11, and 15), suggesting that all of them contain full arrays of Zn fingers.

The biotinylated oligos representing binding sites are indicated above each group of reactions. The table lists the specific material loaded into the lanes.

| Lanes | Material loaded into each lane |
|---|---|
| 1, 5, 9, 14 | Biotinylated oligo only |
| 2, 6, 10, 15 | Biotinylated oligo + PRDM9$^{Dom2}$ |
| 3, 7, 11, 16 | Biotinylated oligo + PRDM9$^{Cst}$ |
| 4, 8, 12, 17 | Biotinylated oligo + induced empty vector |
| 13, 18 | Induced PRDM9 allele (indicated above the lane) directly loaded on the gel (positive control) |

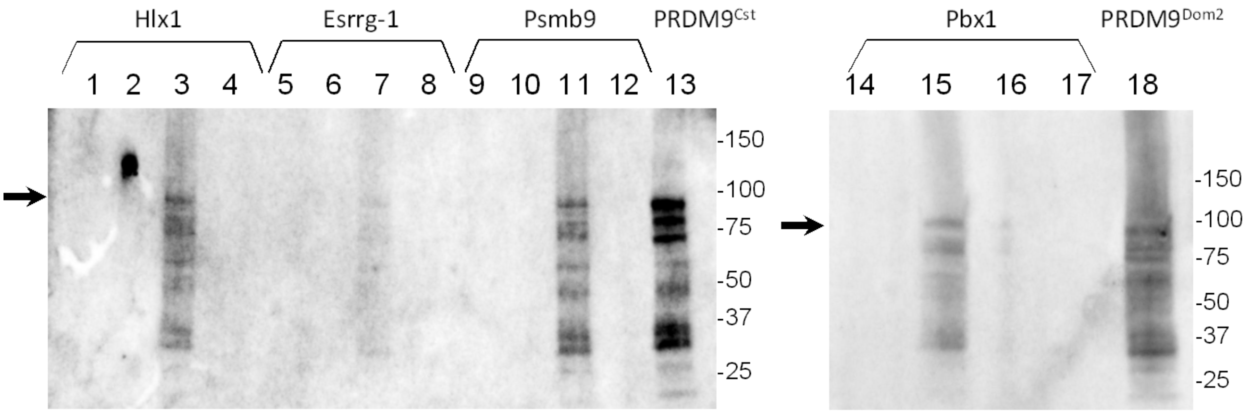

Supplement: Additional file 13 — Figure S8. PRDM9 binds to its targets in an allele-specific manner. The Additional material contains maps of all hotspots studied in this paper, their sequences, additional figures and tables highlighting specific points in the paper, and the sequences of the oligos used for mapping. [file gb-2013-14-4-r35-S13.PDF]
